# BayesFlow: latent modeling of flow cytometry cell populations

**DOI:** 10.1186/s12859-015-0862-z

**Published:** 2016-01-12

**Authors:** Kerstin Johnsson, Jonas Wallin, Magnus Fontes

**Affiliations:** Centre for Mathematical Sciences, Lund University, Box 118, Lund, S-221 00 Sweden; Mathematical Sciences, Chalmers and University of Gothenburg, Gothenburg, S-412 58 Sweden; International Group for Data Analysis, Institut Pasteur, 25 Rue du Docteur Roux, Paris, 75015 France

**Keywords:** Bayesian hierarchical models, Flow cytometry, Model-based clustering, Primary 62P10, Secondary 62F15, 68U99

## Abstract

**Background:**

Flow cytometry is a widespread single-cell measurement technology with a multitude of clinical and research applications. Interpretation of flow cytometry data is hard; the instrumentation is delicate and can not render absolute measurements, hence samples can only be interpreted in relation to each other while at the same time comparisons are confounded by inter-sample variation. Despite this, most automated flow cytometry data analysis methods either treat samples individually or ignore the variation by for example pooling the data. A key requirement for models that include multiple samples is the ability to visualize and assess inferred variation, since what could be technical variation in one setting would be different phenotypes in another.

**Results:**

We introduce BayesFlow, a pipeline for latent modeling of flow cytometry cell populations built upon a Bayesian hierarchical model. The model systematizes variation in location as well as shape. Expert knowledge can be incorporated through informative priors and the results can be supervised through compact and comprehensive visualizations.

BayesFlow is applied to two synthetic and two real flow cytometry data sets. For the first real data set, taken from the FlowCAP I challenge, BayesFlow does not only give a gating which would place it among the top performers in FlowCAP I for this dataset, it also gives a more consistent treatment of different samples than either manual gating or other automated gating methods. The second real data set contains replicated flow cytometry measurements of samples from healthy individuals. BayesFlow gives here cell populations with clear expression patterns and small technical intra-donor variation as compared to biological inter-donor variation.

**Conclusions:**

Modeling latent relations between samples through BayesFlow enables a systematic analysis of inter-sample variation. As opposed to other joint gating methods, effort is put at ensuring that the obtained partition of the data corresponds to actual cell populations, and the result is therefore directly biologically interpretable. BayesFlow is freely available at GitHub.

**Electronic supplementary material:**

The online version of this article (doi:10.1186/s12859-015-0862-z) contains supplementary material, which is available to authorized users.

## Background

In a flow cytometer a number of characteristics for each individual cell in a sample of ∼ 10^4^ to ∼ 10^6^ cells are quantified as they pass through the cytometer in a fluid stream. The data that are obtained are most often summarized by grouping cells into cell populations; properties of these cell populations are used in many clinical applications—for example monitoring HIV infection and diagnosing blood cancers—and in many branches of medical research [1, 2]. Defining the cell populations based on the measured characteristics is in state-of-the-art analyses still done manually by trained operators looking at two-dimensional projections of the data. The importance of automated methods has risen along with an increase of the dimension of typical flow cytometry data sets due to developments in flow cytometry technology [3] and the emergence of studies with large numbers of flow cytometry samples [4]. Furthermore, manual so called gating of cell populations is a subjective process where operators have to take more or less arbitrary decisions for example when there are overlapping populations [5].

Automatic cell population identification is hard since flow cytometry measurements are not absolute, while at the same time different samples cannot be directly compared due to technical variation—especially apparent when samples are analyzed at different laboratories [5]—and intrinsic biological variation within and between subjects. Despite this, research into automated population identification methods has focused on individual or pooled flow cytometry samples, sometimes attempting to align data at first through normalization procedures [6].

Automated methods with the aim to replace manual gating must be able to treat multiple samples jointly and take variation between samples into account, while at the same time make it possible for the user to monitor that variation so that it is not too high for the application at hand. For example it needs to be decided if a shift in location of a population in a sample can be seen as technical variation and accepted or if the changed marker expression means that it is a different cell phenotype. These kinds of methods also need to be able to take prior information into account—in manual gating the experience of the operator can be necessary to define a population. We have developed BayesFlow, a method which models variation in cell population location as well as shape, can include prior information for example about cell population location, and gives a result that can be assessed in compact and comprehensive visualizations.

Partitioning the cell measurements in a sample into cell populations is essentially a clustering problem. In the context of flow cytometry data analysis clustering is called automated gating, as opposed to the manual gating performed by operators. Model-based clustering using mixture models has been the most used approach for automated gating [7–12]. Mixture models are very well suited to describe flow cytometry data because they have a natural biological interpretation based on the cell populations. Examples of other approaches that have been used for automated gating are grid based density clustering [13], spectral clustering [14], hierarchical clustering [15, 16] and k-means clustering [17, 18]. An evaluation of a wide range of automated gating methods was performed in the FlowCAP I challenge [19]. The discrepancy with manual gating was often quite large even for the best methods, with average F-measures around 0.9 for both completely automated and manually tuned methods. Large discrepancies between manual and automatically gated samples can be acceptable since the arbitrary decisions taken in manual gating means that the gates could just as well have been set another way. However, it is important that the gating is consistent between samples so that they can be compared against each other.

Joint identification of cell populations in a collection of samples can be accomplished by pooling the samples [12, 15] or matching populations identified separately in the samples [10, 20]. However, in the first approach no variation between samples is taken into account and in the second approach no information is shared between samples. Recently a third approach has been explored, where a Bayesian hierarchical model is used to share information between samples while at the same time allowing for variation. This was first utilized for flow cytometry gating by Cron et al. [21], with a hierarchical Dirichlet process model with fixed locations and shapes of cell populations. An extension of this model, also modeling variation in cell population locations has been used to create ASPIRE, a method for anomalous sample detection [22].

BayesFlow follows this third approach, but use a differently structured model than what has been used previously, favoring explicit modeling instead of implicit, parametric instead of non-parametric (or massively parametric). This follows the philosophy that mathematical models can never perfectly fit reality, thus it is important to be able to convey the constructed model and its parameters and in what ways it simplifies the data.

For example, in addition to variation in location BayesFlow explicitly models variation in cell population shape, whereas ASPIRE models shape variations implicitly by combining Gaussian components with the same shape. This means that an aberrant shape variation of a cell population in a sample can be detected in BayesFlow by examining the parameters of the model, which is not possible in ASPIRE. Perhaps more importantly, BayesFlow gives a parsimonious model which much fewer parameters—each individual parameter for the components in BayesFlow can be assessed through compact visualizations and thus undesired behaviors can be detected and corrected for by change of setup. Moreover, a restriction in ASPIRE which is avoided by BayesFlow is that the variation of component location within and between samples is connected to the shape of the components.

In BayesFlow, the cells in a sample are clustered using a multivariate Gaussian mixture model (GMM), where *K* components describe true and artificial cell populations and one component describes outliers. Artificial cell populations are measurements that cluster together and behave otherwise like real cell populations, but arise for example from dead cells, non-specific binding of markers or doublets; doublets are pairs or groups of cells that pass through the flow cytometer at the same time. Measurements which are not clearly grouped but spread out over the measurement space, for example due to measurement noise, are modeled as outliers.

For each component not representing outliers its mean and covariance matrix is linked to a latent cluster which collects corresponding components across all samples. In practice this is done by assuming a normal prior for the means and an inverse Wishart prior for the covariance matrices of the components linked to a given latent cluster. The parameters of sample and latent components are jointly estimated by Markov Chain Monte Carlo (MCMC) sampling. The variation in location and shape between corresponding mixture components across samples is controlled by the priors on parameters of the latent clusters. The location of component means and shape of components can also be restricted if there is prior information supporting this. To allow for that flow cytometry data frequently have missing cell populations, we include the possibility that not all components are present in every sample.

A challenge that has to be addressed when analyzing flow cytometry data is that cell populations can be skewed and/or have heavy tails and are then not well described by a single Gaussian component [7, 10, 23]. To handle this we use multiple components to model such populations, an approach that have often been employed for flow cytometry data [9, 12, 24, 25] and has the further advantage that the number of cell populations can be automatically detected. We merge Gaussian components into super components with a procedure based on a systematic study of methods for merging mixture components [26].

Results from the MCMC sampling and subsequent merging are evaluated in a number of quality tests. This is a crucial step since what is deemed as a good clustering is application dependent. In some settings a given amount of variation in location or shape is expected from biological or technical reasons, whereas in others the same variation would indicate a different population. This also means that it is necessary for the user to choose prior parameters for their application. To simplify this process we have derived parametrizations so that the same value of the parameters gives a similar effect of the prior on data sets of different sizes.

We verified the ability of the sampling scheme to recover model parameters by fitting the model to a small three-dimensional synthetic data set with 1.2 million cells in total and a large synthetic data set with in total 28 million cells in 8 dimensions. Then we applied BayesFlow to one of the datasets in the FlowCAP I challenge, the GvHD dataset, which contains samples from patients who have had organ transplants and might have early signs of graft-versus-host disease. We show that BayesFlow does not only give a result which has the same degree of accordance with manual gating as the best performing methods in FlowCAP I—which is much higher than what is obtained for other methods based on joint gating with Bayesian hierarchical models—it does also give a more similar treatment of different samples than manual gating and the best methods from FlowCAP I. Finally we applied BayesFlow, ASPIRE [22] and HDPGMM [21] to a data set with replicated samples from four healthy individuals. The ratio between intra-donor technical variation and inter-donor biological variation was similar between BayesFlow and HDPGMM, which was lower than for ASPIRE. Moreover, BayesFlow was the only of the three methods which gave cell populations with clear expression patterns.

## Methods

### Model

Let ***Y***_*ij*_ denote vector valued measurement number *i* in sample *j*. Here *i*∈{1,…,*n*_*j*_}, where *n*_*j*_ is the number of cells in sample *j*, and *j*∈{1,…,*J*}, where *J* is the number of samples. We let the dimension of the observations be denoted *d*. With *K* mixture components describing cell populations the probability density for cell measurement *i* of a flow cytometry sample *j* is modeled as

(1) $$ f(\mathbf{Y}_{ij}) = \sum\limits_{k = 1}^{K} \pi_{jk}N\left(\mathbf{Y}_{ij}; \boldsymbol{\mu}_{jk}, \Sigma_{jk}\right) + \pi_{j0}N\left(\mathbf{Y}_{ij};\boldsymbol{\mu}_{j0},\boldsymbol{\Sigma}_{j0}\right),  $$

where *N*(**Y**;***μ***,***Σ***) denotes the probability density function of the normal distribution with mean ***μ*** and covariance matrix ***Σ*** evaluated at **Y**. To facilitate interpretation, the number *K* should be chosen as small as possible, given that the model pass quality requirements (described under Quality control). The last component represents outliers and its parameters ***μ***_*j*0_=***μ***_0_ and ***Σ***_*j*0_=***Σ***_0_ are identical across samples. Outliers are often modeled by a uniform density over the measurement space [27]; however due to the curse of dimensionality [28], this is not well behaved when we have more than a few dimensions, in which case a Gaussian should perform better. Noise coming from for example dead cells can also be captured in artificial cell populations, and can be excluded in downstream analyses based on the expression patterns.

The vector ***π***_*j*_={*π*_*j*0_,…,*π*_*jK*_} contains the mixing proportions, i.e. the proportion of cells described by the component. To connect cell populations between samples we use a latent layer, assuming that for a given *k* each ***μ***_*jk*_ and ***Σ***_*jk*_ is drawn from a normal and an inverse Wishart distribution respectively. Specifically, in our model, for *k*=1,…,*K*,

(2) $$ \begin{aligned} \boldsymbol{\mu}_{jk} | \boldsymbol{\theta}_{k}, \boldsymbol{\Sigma}_{\theta_{k}} \sim N(\boldsymbol{\theta}_{k}, \boldsymbol{\Sigma}_{\theta_{k}}), \\ \boldsymbol{\Sigma}_{jk}| \boldsymbol{\Psi}_{k},\nu_{k} \sim IW(\boldsymbol{\Psi}_{k},\nu_{k}) \end{aligned}  $$

where ***θ***_*k*_, $\boldsymbol {\Sigma }_{\theta _{k}}$, ***Ψ***_*k*_ and *ν*_*k*_ are hyper-parameters describing latent cluster *k*. These parameters describes the variability between flow cytometry samples, in contrast to ***μ***_*jk*_,***Σ***_*jk*_ which describe the distribution of cell measurements within a sample. The normal and inverse Wishart distributions are conjugate priors to the mean and the covariance respectively of the normal distribution, enabling efficient sampling, however they are not jointly conjugate.

We call ***θ***_*k*_ and ***Ψ***_*k*_/(*ν*_*k*_−*d*−1) the latent cluster mean and latent cluster covariance matrix respectively, since they are the a priori expected values of ***μ***_*jk*_ and ***Σ***_*jk*_.

**Fig. 1:**
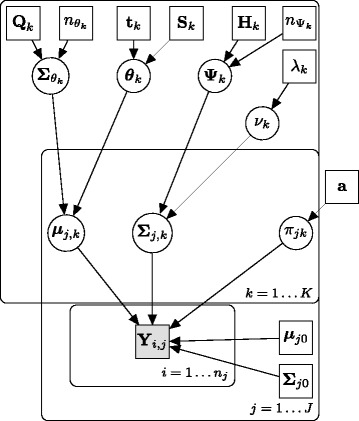
Directed acyclic graph describing the Bayesian hierarchical model. Square boxes indicate that the values are known

For the hyper-parameters describing the latent clusters and the mixing proportions we use the following prior distributions:

(3) $$\begin{array}{*{20}l}  \boldsymbol{\theta}_{k} | \mathbf{t}_{k}, \mathbf{S}_{k} &\sim N(\mathbf{t}_{k}, \mathbf{S}_{k}), & \boldsymbol{\pi}_{j} &\sim \, D(\mathbf{a}),\\ \boldsymbol{\Sigma}_{\theta_{k}}|\mathbf{Q}_{k},n_{\theta_{k}} &\sim IW(\mathbf{Q}_{k},n_{\theta_{k}}), & \nu_{k}|\lambda_{k} &\sim \exp(-\lambda_{k}),  \\ \boldsymbol{\Psi}_{k} | \mathbf{H}_{k},n_{\Psi_{k}} &\sim W(\mathbf{H}_{k},n_{\Psi_{k}}), & & \end{array} $$

where *W* denotes the Wishart distribution and *D* denotes the Dirichlet distribution, which is conjugate prior to the multinomial distribution. For each *ν*_*k*_ we assign a exponential prior on the positive natural numbers. The complete structure of the model is displayed through a directed acyclic graph (DAG) in Fig. 1.

The parameters **t**_*k*_ and **S**_*k*_ define the prior belief of the locations of the latent means ***θ***_*k*_, whereas the parameters **Q**_*k*_ and $n_{\theta _{k}}$ control the spread of mixture component means within a latent cluster and are hence important to control the variation across samples. A large $n_{\theta _{k}}$ along with a small **Q**_*k*_ forces the ***μ***_*jk*_ together; it makes large deviations between $\boldsymbol {\Sigma }_{\theta _{k}}$ and **Q**_*k*_ unlikely. The parameters **H**_*k*_ and $n_{\Psi _{k}}$ control the expected values and the variation of latent covariance matrices as well as the variation among mixture component covariance matrices in a latent cluster. If $n_{\Psi _{k}}$ is large each ***Σ***_*jk*_ will be close to ***Ψ***_*k*_/(*ν*_*k*_−*d*−1) for any *k*, since a high $n_{\Psi _{k}}$ makes high *ν*_*k*_ more probable.

Finally, to simplify sampling from the posterior distribution of the parameters, we add an component assignment variable *x*_*ij*_∈{0,1,…,*K*} describing which component **Y**_*ij*_ is drawn from. To comply with (1), the a priori uncertainty of component membership is modeled by *x*_*ij*_∼ *Mult*(***π***_*j*_,1), where *Mult* denotes the multinomial distribution.

The resulting posterior distribution of all the parameters, denoted jointly by ***Θ***, and ***x*** given the data **Y** is given in the Additional file 1: Section A. In Section B we describe the Markov chain Monte Carlo (MCMC) sampling scheme used to generate posteriors for our model parameters.

The computational bottleneck of the sampling scheme is the sampling of x, with a computational complexity bounded by $\mathcal {O} (J d^{3} K\max _{j} n_{j})$. To handle high dimensions diagonal covariance matrices can be used instead, in which case the complexity is bounded by $\mathcal {O}(J d K\max _{j} n_{j})$. However, for datasets with more than 20 dimensions the mathematical feasibility of using Gaussian mixture models without any prior dimension reduction needs to be seriously considered first, due to the curse of dimensionality [28].

#### Absent components

In some flow cytometry data sets not all cell populations are present in all samples. In our model this corresponds to that *π*_*jk*_=0 for some (*j*,*k*). However, mixture component parameters for empty clusters will still affect the mixing of the MCMC for the parameters of the latent cluster. It can also happen that if a cluster is empty that the mixture component moves and split a neighboring cluster in two. To avoid this in such data sets we extend the model by introducing a variable **Z**_*j*_∈{0,1}^*K*^ that says which components are active in sample *j*. This has the further advantage that when sampling from the posterior distribution of the model we get the probability for each cluster that it is present in a sample. We impose a prior on **Z**_*j*_ which is proportional to $\exp \left (-c_{s}\sum _{k=1}^{K}\mathbf {Z}_{j}\right) I\left (\sum _{k=1}^{K}\mathbf {Z}_{j}>0\right)$ where *I* denotes the indicator function and *c*_*s*_>0. The prior makes the model prefer fewer activated clusters so that if there is a very small cluster the likelihood will be larger if it is inactivated, which prevents spurious clusters. The strength of this prior can be adjusted to the expected size of the smallest clusters.

The changes to (1–3) required by this extension are straightforward but inference of the model becomes a bit more involved since removing components reduces the dimension of the model. To accommodate for this we have included a reversible jump step in our sampling algorithm. Details are given in the Additional file 1: Section B.

### Merging latent clusters

To determine the “correct” number of clusters in a data set directly from the data is an ill-defined problem, since what should be considered to be a separate cluster depends on the interpretation of the data. Nevertheless, there are many different criteria which can be used to guide the decision about the number of populations [26, 29]. We use overlap between components—measured by Bhattacharyya distance—and unimodality of the resulting super clusters—measured by Hartigan’s dip test [30]—to determine which latent clusters to merge and to indicate our confidence in the mergers.

In an evaluation of criteria for merging Gaussian components to represent more complex distributions, the Bhattacharyya distance performed well [26]. Bhattacharyya distance merges clusters according to a pattern-based cluster concept as opposed to a modality-based concept [26]. With a pattern-based cluster concept a small dense cluster inside a sparse cluster—for example a well specified cell population inside a region with sparse outliers—will be considered to be different clusters. This would not be the case for the modality-based cluster concept as long as the generating probability density is unimodal.

The Bhattacharyya distance between *N*(***μ***_1_,***Σ***_1_) and *N*(μ_2_,***Σ***_2_) is

(4) $$ \begin{aligned} d_{\text{bhat}} = 1/8 \cdot (\boldsymbol{\mu}_{1} - \boldsymbol{\mu}_{2})^{\top} \bar{\boldsymbol{\Sigma}}^{-1} (\boldsymbol{\mu}_{1} - \boldsymbol{\mu}_{2}) \\+ 1/2 \cdot \log \left(|\bar{\boldsymbol{\Sigma}}| / \sqrt{|\boldsymbol{\Sigma}_{2}||\boldsymbol{\Sigma}_{2}|} \right), \end{aligned}  $$

where $\bar {\boldsymbol {\Sigma }} = (\boldsymbol {\Sigma }_{1} + \boldsymbol {\Sigma }_{2})/2$ [31]. In order to measure Bhattacharyya distance between mixtures of Gaussian distributions, which is necessary for deciding if super clusters should be merged with other clusters, we approximate each mixture with a Gaussian distribution. The means and the covariance matrices are estimated using a soft clustering of the data points inferred from the sampling of *x*_*ij*_, detailed in the Additional file 1: Section C.

However, it is not obvious how to set a threshold for *d*_bhat_, since the appropriate threshold depends on the distribution of the data [26], which is unknown. Because of this we use a low soft threshold *d*_1_ and a high hard threshold *d*_2_. Two clusters closer to each other than *d*_1_ are always merged, two clusters whose distance is between *d*_1_ and *d*_2_ are only merged if they fulfill an additional criterion based on Hartigan’s dip test for unimodality.

Unimodality is an appealing heuristic for defining cell populations, and it has frequently been used for automated gating [9, 12, 18]. It has two main limitations. The first one, that populations intuitively should be separate if they have very different densities—even when they overlap so that their combined distribution is unimodal—can be bypassed by combining unimodality with a pattern-based merging criterion such as Bhattacharyya distance. The second one, that it is difficult to determine if a multi-dimensional empirical distribution is multimodal, is usually handled by considering one-dimensional projections [12, 26]. This is the approach we take here, using Hartigan’s dip test of unimodality for each of the projections onto the coordinate axes where Bhattacharyya overlap is low, and for the projection onto Fisher’s discriminant coordinate. If for a proposed merger, any of these projections is found to be multimodal, the clusters are not merged. Further details of the merging procedure are given in the Additional file 1: Section C.

### Quality control

To verify that the output of BayesFlow fulfills the user’s requirements, a number of checks are performed:

- Convergence of the MCMC sampler is established by viewing trace plots of sampled parameters.
- To ensure that variation of the two different populations are not confused with each other, we require that the Bhattacharyya distance as well as the Euclidean distance from each sample component to its corresponding latent component should be smaller than these distances to any other latent component which does not belong to the same super cluster.
- To ensure that the obtained clusters should not be divided further, Hartigan’s dip test is computed for the projections onto the coordinate axes of all super clusters. Projections which have a dip test p-value below 0.28—the threshold for merging components (see Additional file 1: Section C)—are visualized using histograms of quantiles of the weighted data belonging to the cluster.
- To ensure that the model fits the data reasonably well, samples from the posterior predictive is compared to the true data in one- and two-dimensional histograms.
- To ensure that there are no outliers among the cluster centers, the centers for each cluster are plotted together along one dimension.
- Additionally, to detect components with aberrant shapes, the eigenvectors corresponding to the largest eigenvalues, multiplied with the corresponding eigenvalues, can be viewed.

If any of the quality criteria is not met, the simulation should be rerun, either using the same or different parameters. Even if the same parameters are used a different result can be obtained due to randomness in the initialization.

### Experiments

#### Simulated data

In order to verify that the proposed sampling scheme can find the correct model parameters, the MCMC algorithm was applied to two simulated datasets. The first dataset was three-dimensional, which enables direct visual evaluation. It had four latent clusters across eighty artificial flow cytometry samples; each sample had 15,000 cells giving a total of 1.2 million cells. One of the latent clusters was present only in eight samples and another one was present in 24 samples, so that the ability to find rare cell populations was tested. Moreover, the cluster which was present in only eight samples contained only 1 *%* of the total number of cells, thus also the ability to find small cell populations was tested. The parameters and the algorithm used for generating the data are given in the Additional file 1: Section D.1.

The second data set was designed to test the ability to handle large data. It was eight-dimensional, with eleven latent clusters and 192 artificial flow cytometry samples. Each sample had measurements of 150,000 cells, giving a total of 28 million cells. Four of the eleven clusters were missing in half of the samples. Additional file 2 contains a python script and data for regenerating this data set.

Prior parameters and initial values for the MCMC sampler are given in the Additional file 1: Section D.1. All priors were chosen to be non-informative. The outlier component was not used for inference in the small dataset, but it was used for the large dataset. The MCMC sampler ran first for a number of burn-in iterations, then the posterior distribution was explored in a number of production iterations. During the production iterations, apart from sampling parameters of the model, a value of ***Y*** was also drawn, i.e. a sample from the posterior predictive. For the first synthetic data set 10,000 burn-in and 100,000 production iterations were used. For the second, larger, data set we used 5,000 burn-in iterations and 5,000 production iterations.

For the second data set the MCMC sampler was run on Amazon Cloud, using 192 cores. Each iteration took on average one second, so that about 2.7 h was needed in total.

#### Flow cytometry data

We analyze two flow cytometry data sets with BayesFlow: the data set GvHD from the FlowCAP I challenge—with four markers, 12 samples and approximately 13,000 cells per sample—and a data set obtained from the R package healthyFlowData [32] with technical replicates of PBMC samples from healthy donors—in total 20 samples with approximately 20,000 cells, also measured with four markers. In the GvHD dataset we can compare the gating obtained from BayesFlow with manual gating provided from FlowCAP as well as automated gating from a wide range of other methods. In healthyFlowData we can instead compare gating between technical replicates to see if samples are treated in a consistent manner.

For the healtyFlowData dataset we used an exploratory approach with non-informative priors. We ran multiple simulations and gradually increased the number of components until we passed the quality criteria described under Quality control; we finally arrived at using *K*=25 components. For the GvHD data set we started with an exploratory approach and gradually increased the number of components, but in the quality checks we noted one population in one of the samples which was very hard to capture. Then we decided to use an informative approach for this population. Using a scatter plot, Fig. 6, we set boundaries for this population in the dimensions given by the CD4 and the CD8b marker and computed its mean and empirical s covariance matrix. We used the mean to set an informative prior for ***θ***_*k*_ and the mean and the empirical covariance to initialize the component. Prior parameters in both the informative and non-informative case are described in the Additional file 1: Section E.2.

BayesFlow applies three data preprocessing steps: 1) Data points with extreme values in at least one dimension (larger than 0.999 times the largest data point or smaller than 1.001 times the smallest data point) are removed. Such data points can lead to components with singular covariance matrices, and a well designed flow cytometry experiment should not have significant populations with such values. 2) The data is scaled using the 1 and 99 % percentiles *q*_0.01_ and *q*_0.99_ of the pooled data, with the same scaling for all samples, so that *q*_0.01_=0 and *q*_0.99_=1 for each marker for the pooled data. This is done in order to be able to set informative priors in an intuitive way. 3) Before testing which components should be merged, a very small amount of noise is added to the data (standard deviation 0.003). This is since the discreteness of the original flow cytometry measurements can lead to a striped pattern in the flow cytometry data [33] and also when it is not visible to the human eye it disturbs the dip test.

After preprocessing, parameters for the MCMC sampler were initialized by running the EM algorithm on the pooled data, followed by the initialization scheme used for the large synthetic dataset, detailed in the Additional file 1: Section D.4. We ran 16,000 burn-in iterations and 4,000 production iterations of the MCMC sampler for both experiments. The burn-in period consisted of five phases: In the first phase, the priors on variation in location and shape were modified to force clusters together. Before the second phase, priors parameters were set to normal again. After the second phase, components which were considered to be outliers were turned off. They were forced to stay off during a short third phase, but from the forth phase and onwards components were allowed to turn on and off. Label switching was allowed during the initial four phases in order to escape non-desired local minima, but then disallowed. The values of parameters controlling the simulation during the burn-in and production period are given in Additional file 1: Table S1.

We also applied the two other joint gating methods based on Bayesian hierarchical models: ASPIRE [22] and HDPGMM [21]. For ASPIRE parameters were chosen according to the strategy recommended by Dundar et al. [22]; details are given in the Additional file 1: Section E.5. For each run we used in total 15,000 iterations, of which 14,000 were set as burn in iterations. For HDPGMM default parameters were used, with a burn-in period of 3,000 iterations and a production period of 100 iterations.

We ran BayesFlow and ASPIRE on a 3.2 GHz quad core CPU. A BayesFlow run took 0.5 h for the GvHD dataset and 1.4 h for healthyFlowData. ASPIRE took in total 2.4 h for the GvHD dataset and 6.6 h for healthyFlowData per run. Four runs of ASPIRE was needed to determine the *κ*_*i*_ parameters. HDPGMM was run on a dual core GPU. It needed 0.72 h for the GvHD dataset and approximately 1 h for the healthyFlowData dataset.

## Results

### Simulated data

**Fig. 2:**
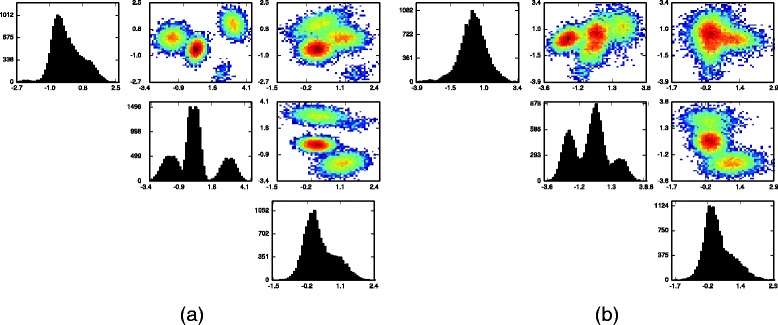
**a** One and two dimensional histograms for one synthetic flow cytometry sample containing 15,000 data points; **b** histograms of 15,000 data points drawn uniformly from the pooled data from the synthetic data experiment

We begin by analyzing the smaller data set. In Fig. 2 we show univariate and bivariate histograms of all synthetic cell measurements pooled together, as well as the corresponding histograms of the data from a single flow cytometry sample where all four clusters are present. Note that the data when pooled together has a complicated density, as it is in fact a mixture of 232 multivariate normal densities.

**Fig. 3:**
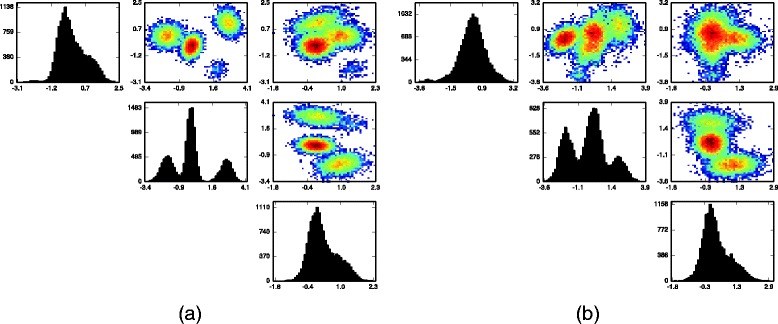
**a** One and two dimensional histograms of 15,000 posterior draws of ***Y*** for the flow cytometry sample displayed in Fig. 2 **a**; **b** histograms of 15,000 posterior draws of ***Y*** drawn uniformly from all the flow cytometry samples, thus matching Fig. 2 **b**

In Fig. 3 we show the same univariate and bivariate histograms, but this time with samples from the posterior predictive distribution of ***Y***. From the synthetic cell measurements generated from the inferred models of the datasets it is clear that the inferred models are accurate and capture the variation across samples, which a model only of pooled data cannot do.

**Fig. 4:**
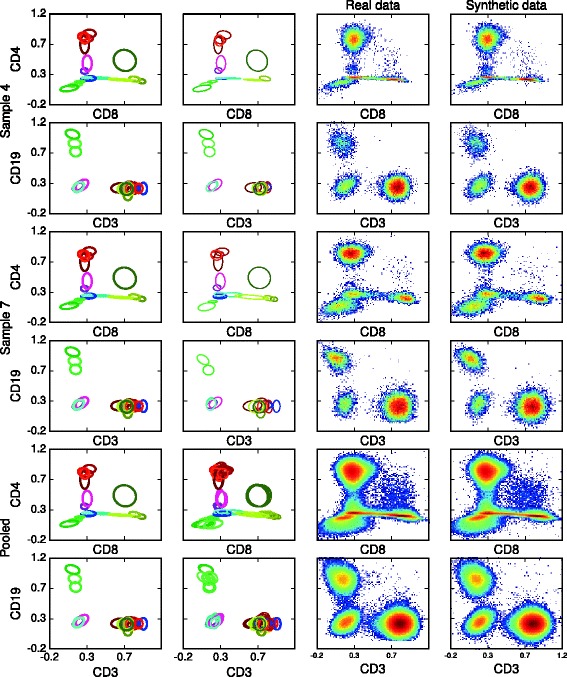
BayesFlow component parameter representations of inferred latent clusters (*first* column) and mixture components (*second* column) together with histograms of real data (*third* column) and synthetic data generated from the model (*fourth* column) for healthyFlowData. The center of each ellipse is the mean and each semi-axis is an eigenvector with length given by the corresponding eigenvalue of the projected covariance matrix. For the latent clusters the parameters $(\boldsymbol {\mathrm {\theta }}_{k},\frac {1}{(\nu _{k}-d-1)}\boldsymbol {\mathrm {\Psi }}_{k})$ are shown, for the mixture components the parameters (***μ***_*jk*_,***Σ***_*jk*_) are shown. Each component or cluster is depicted with the same color as in Fig. 5; different shades of same color corresponds to latent clusters that have been merged

**Fig. 5:**
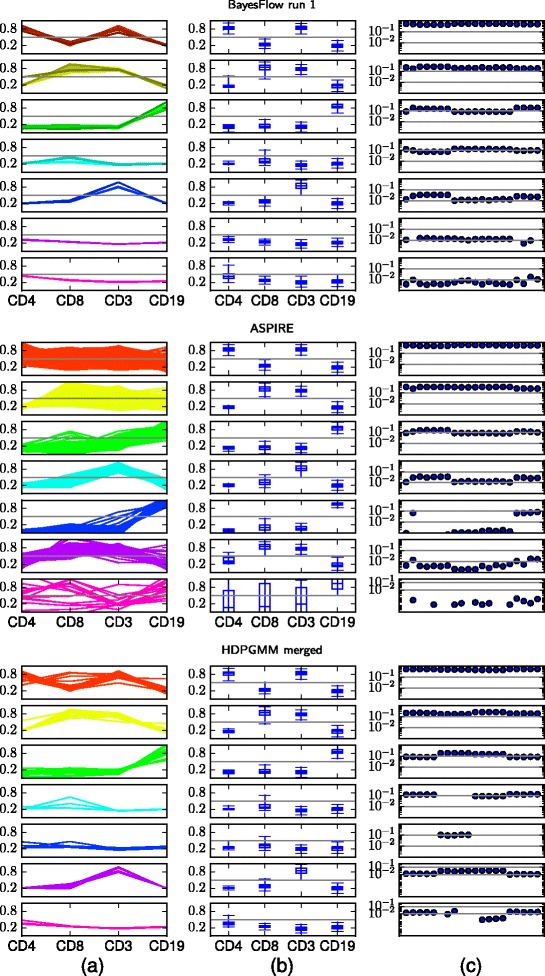
Summary statistics of inferred cell populations in BayesFlow, ASPIRE and HDPGMM, ordered by population size. For HDPGMM, the six largest components after merging are shown, the remaining components have together at most 0.0013 of the cells in a sample. The noise component in BayesFlow has at most 0.004 of the cells in a sample. **a** Locations ***μ***_*jk*_ of mixture components that represent each population, in each sample, cf. Fig. 13. **b** Box plots of the soft clusters in the pooled data, cf. Fig. 13. **c** Population proportions across flow cytometry samples

**Fig. 6:**
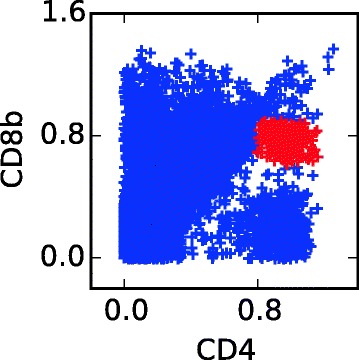
Cell population which is hard to detect in the GvHD dataset

**Fig. 7:**
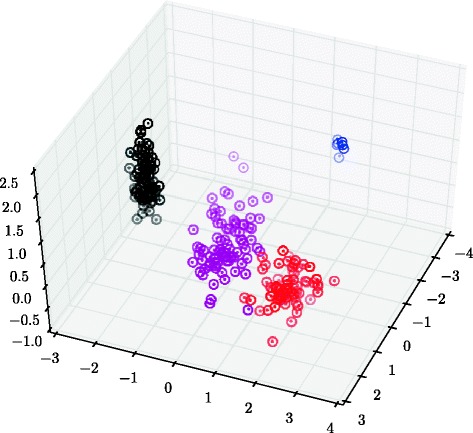
The posterior mean of the mixture component centers, ***μ***_*jk*_ (*dots*), and the true cluster centers (*circles*) in the small synthetic data experiment

Figure 7 displays dots at the posterior mean locations of the mixture component centers ***μ***_*jk*_ whose posterior probability of being active is greater than 1 *%*; the true locations of the active clusters are displayed as circles. The model is able to detect which clusters that are active and which are not, and to find the location of the component means.

**Fig. 8:**
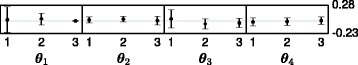
The difference between the true value of each entry in each ***θ***_*k*_ and the approximated marginal posterior distribution generated by the MCMC sampler in the small synthetic data experiment. The black dot represents the median and the vertical line goes between the 2.5 and 97.5 *%* quantiles. The light gray horizontal line is the 0 line

**Fig. 9:**
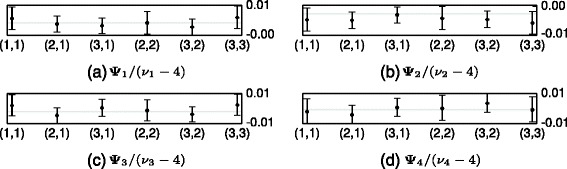
The difference between the true value of each of the entries in ***Ψ***_*k*_/(*ν*_*k*_−4) and the approximated marginal posterior distribution generated by the MCMC sampler in the synthetic data experiment. The black dot shows the median, and the black vertical line goes between the 2.5 and 97.5 *%* quantiles. The light gray horizontal line is the 0 line

Finally in Figs. 8 and 9, the marginal posterior distributions of the latent cluster parameters ***θ***_*k*_ and ***Ψ***_*k*_, subtracted by their true values, are presented. In Fig. 8 the dot represents the difference between the median of posterior distribution and the true value of each ***θ***_*k*_. The vertical lines represent the 2.5 and 97.5 *%* quantiles. Fig. 9 displays results for each latent covariance matrix ***Ψ***_*k*_/(*ν*_*k*_−4) in the same way. From Figs. 8 and 9 we see that the true parameters of both the means and the covariances are all between the 2.5 and 97.5 % quantiles of the posterior distribution.

**Fig. 10:**
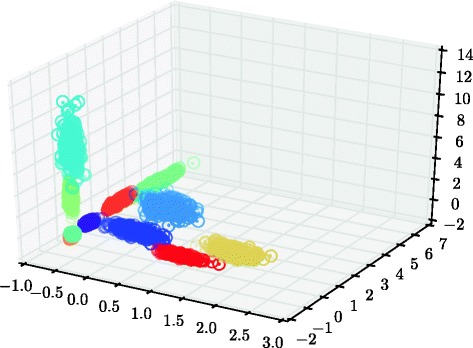
The posterior mean of the mixture component centers, ***μ***_*jk*_ (*dots*), and the true cluster centers (*circles*) in the large synthetic data experiment for the first three dimensions

The true and estimated cluster centers of the eight-dimensional data set cannot be displayed efficiently with just three dimensions at hand, but a three-dimensional projection is shown in Fig. 10. The average error in Euclidean distance in the full eight-dimensional space is 0.007, which can be compared to the average error had the latent mean across samples been used, namely 0.110, which is the best that could have been obtained from a model not including variation between samples. The outlier component was used for inference in the results presented here, but omitting it has very small effect.

**Fig. 11:**
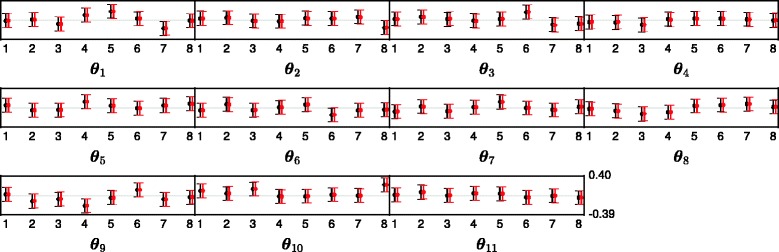
The difference between the true value of each entry in each ***θ***_*k*_ and the approximated marginal posterior distribution generated by the MCMC sampler in the large synthetic data experiment. The black dot represents the median and the vertical line goes between the 2.5 and 97.5 *%* quantiles. To get the axis on the same scale for all the clusters, they are scaled by the standard deviation of ***μ***_*k*_. The light gray horizontal line is the 0 line. The red dot and lines is the same however where one uses the true ***μ***_*k*_ to estimate ***θ***_*k*_, rather then the ***μ***_*k*_ obtained by taking the posterior means of the mixtures

In Fig. 11, we show the posterior distribution of the latent cluster means where again the dot represents the difference between the median of posterior distribution and the true value of each ***θ***_*k*_. The vertical lines are the 2.5 and 97.5 *%* quantiles. The posterior samples have been divided by the standard deviation of the true ***θ***_*k*_ so that the scales across the clusters are equal. Some of the credibility intervals do not contain zero, but this is explained when studying the intervals that would have been obtained if the true ***μ***_*k*_ were used (shown in red), since they are almost identical.

We thus see that cluster centers and credibility intervals for latent clusters are captured well in both synthetic data sets.

### Flow cytometry data

#### GvHD

For the analysis of the GvHD dataset we did twelve runs of BayesFlow in the informed setup described above. Seven were excluded due to confusion between populations, i.e. at least one sample component was closest to the wrong latent component; of the remaining five, one more run was excluded since it has not converged, and another two because of multimodal clusters. This leaved two runs that passed the quality control. Additional file 1: Figs. S2 and S3 show trace plots and projections of clusters with high dip test values respectively.

Table 1 reports the accordance with manual gating for the two BayesFlow runs as well as what is obtained from ASPIRE and HDPGMM, as well as the top two performing methods for this data set in FlowCAP: flowMeans and SamSPECTRAL.

**Table 1:** Accordance with manual gating for GvHD data set. For HDPGMM we also report the result when components are merged according to our merging procedure. When this procedure is applied to the results obtained by ASPIRE, no components are merged, i.e. the original result is identical to what is obtained after mergeing

Method	F-measure	Precision	Recall
BayesFlow run 1	0.91 (0.86, 0.95)	0.96	0.89
BayesFlow run 2	0.87 (0.82, 0.92)	0.95	0.84
ASPIRE	0.67 (0.63, 0.72)	0.86	0.63
HDPGMM	0.35 (0.30, 0.39)	0.98	0.23
HDPGMM merged	0.60 (0.54, 0.66)	0.95	0.48
*flowMeans*	*0.88 (0.82, 0.93)*	*0.93*	*0.86*
*SamSPECTRAL*	*0.87 (0.81, 0.93)*	*0.96*	*0.83*
*Ensemble FlowCAP*	*0.88*		

**Fig. 12:**
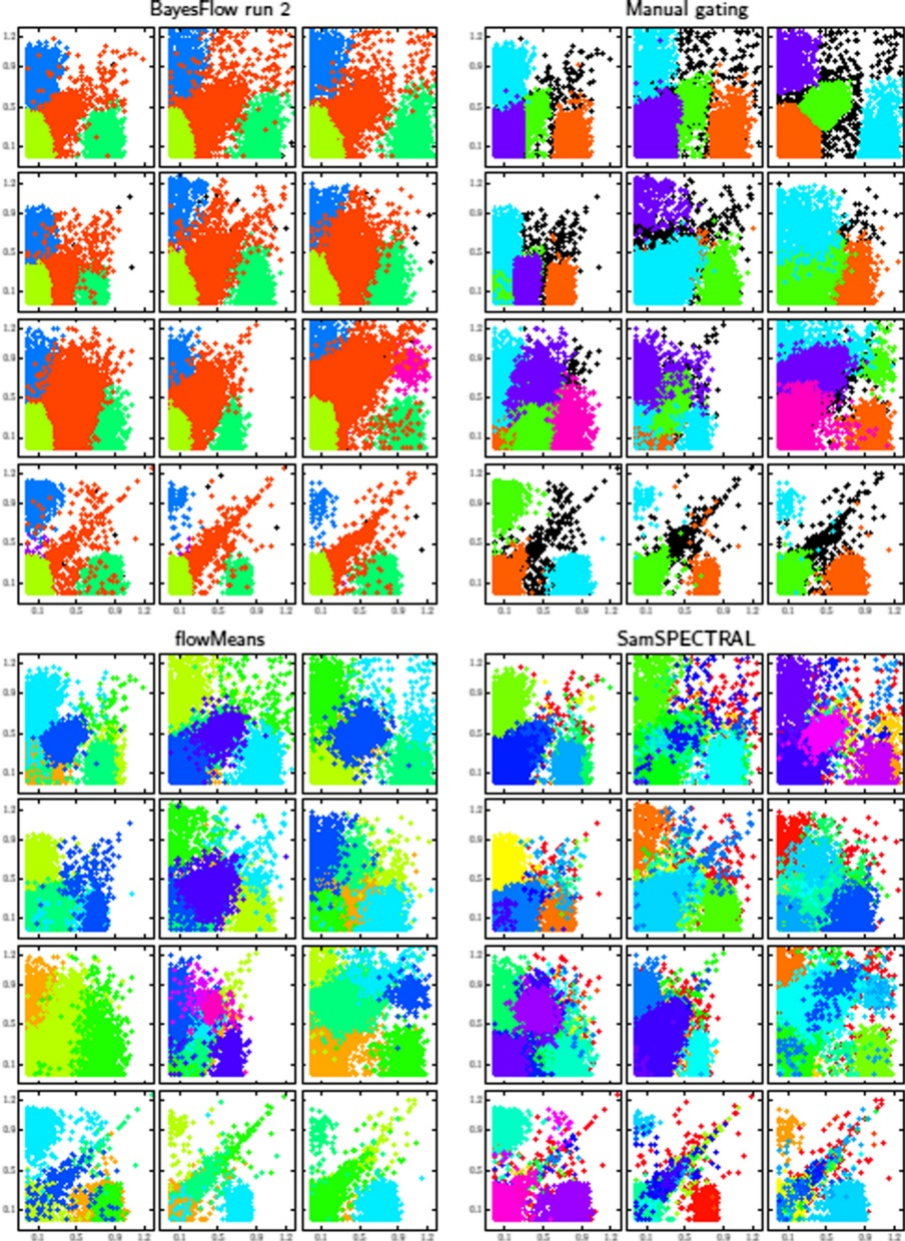
Gated events according to four methods (BayesFlow, manual and the two top performers in FlowCAP I) of the twelve samples in the GvHD dataset, projected onto the two first dimensions. For BayesFlow, the run with least accordance with manual gating, run 2, is shown. Similar plots for ASPIRE and HDPGMM as well as BayesFlow run 1 are shown in the Additional file 1: Figure S6

One of the two BayesFlow runs has the highest accordance with manual gating, the other one is on par with flowMeans and SamSPECTRAL, which is considerably higher than ASPIRE and HDPGMM. However, as can be seen in Fig. 12, the gating of different samples is arguably most consistent for BayesFlow as compared to manual gating, flowMeans and SamSPECTRAL.

**Fig. 13:**
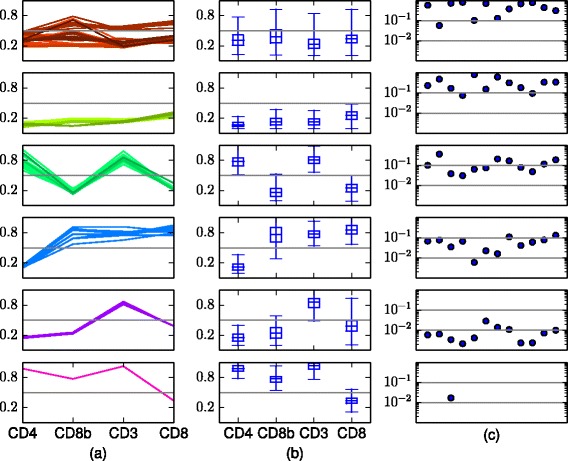
Summary statistics of the six cell populations obtained by BayesFlow (run 2) in the dataset GvHD. The outlier component has at most 0.0019 of the cells in a sample. **a** Each panel displays the locations ***μ***_*jk*_ of all mixture components that represent the population, across all samples. Different shades of a color represent different latent components *k*. **b** Box plots of the soft clusters in the pooled data. The boxes go between the quantiles *q*_*km*,0.25_ and *q*_*km*,0.75_, the whiskers extend to *q*_*km*,0.01_ and *q*_*km*,0.99_. The *α*-quantile for (merged) component *k* in dimension *m*, *q*_*km*,*α*_, is here defined as $q_{km,\alpha } = \min _{i'j'}\{Y_{i'j'm} : \alpha < \sum _{ij:Y_{\textit {ijm}} < Y_{i'j'm}} w_{\textit {ijk}} \}$. **c** Population proportions in each of the twelve flow cytometry samples

To get a further understanding of the variability between samples in BayesFlow, summary statistics for the obtained components and cell populations are shown in Fig. 13.

#### healthyFlowData

We did 18 runs of BayesFlow with *K*=25. Ten of these were excluded due to confusion between populations, moreover two runs were excluded since they had clusters with clearly multimodal distributions. For the six runs that passed the quality control, 3–6 components were turned off across all samples; they are excluded from visualizations. Additional file 1: Figs. S1, S3 and S4 show trace plots, projections of clusters with high dip test values and eigenvectors of covariance matrices respectively.

In Fig. 4 we visualize model fit and inter-sample variation for the first of the six runs that passed the quality control by plotting latent and sample components as well as histograms of real data and synthetic data generated from the model, for two different samples and for the pooled data. We can thus see how shape variations are captured by the model.

The output of BayesFlow, ASPIRE and HDPGMM can be compared in Fig. 5. The merging procedure we used for BayesFlow has been applied for both ASPIRE and HDPGMM, however for ASPIRE no components were merged by this. In BayesFlow each of the populations correspond to clear expression patterns, which is not the case for the other methods. For example the first population is clearly CD4+CD8- T-cells whereas for both ASPIRE and HDPGMM this population contains both components which are CD8- and components which are CD8+.

**Fig. 14:**
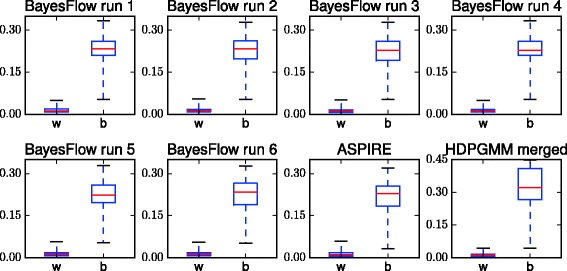
Distances within (*w*) and between (*b*) donors as measured by *ℓ*_1_ distance between vectors of population sizes. For the six BayesFlow runs and HDPGMM there is very little or no overlap between within-donor and between-donor distances, whereas for ASPIRE there is clear overlap

We also compare intra-donor variation of cell population size to inter-donor variation for the six BayesFlow runs, as well as for ASPIRE and HDPGMM in Fig. 14. For ASPIRE there are inter-donor distances which are clearly smaller than some intra-donor distances, which is not the case for BayesFlow and HDPGMM.

## Discussion

From different runs of BayesFlow we can get different representations of data, as in the case of the GvHD dataset. This is because with highly overlapping populations there might be multiple models representing the data equally well. But since all samples are gated jointly in every run, the gated populations can still be compared across samples. The user might have a preference for one representation or the other though, and informative priors can be used to guide BayesFlow to a preferred representation.

BayesFlow is not aimed at discovery of rare cell populations, but it can be used together with an algorithm specifically designed for detecting rare cell populations in a sample, such as SWIFT [12], and then use informative priors to find how this population occurs across an entire set of samples, in a similar way as was done in the GvHD dataset.

How much clusters should be merged is a decision that needs to be taken by the interpreter of the data. In some settings one might want to be restrictive with merging and then use higher thresholds. In others one might want additional mergers after viewing joint one-dimensional projections of the clusters.

The BayesFlow pipeline does not in itself include any compensation or any of the non-linear transformations which are often used for flow cytometry data, such as logicle. Compensation is a linear transformation and Gaussian Mixture Models are invariant under linear transformations, so they perform equally well on uncompensated and compensated data. Non-linear transformations such as logicle can make Gaussian populations non-Gaussian, which makes inference harder. The flow cytometry data we used for the experiments had already been compensated, the healthyFlowData data set had also been transformed with an asinh transform; details are given in the Additional file 1: Section E.1.

BayesFlow finds a joint representation of an entire set of samples. In order for this representation to be reasonable there has to be sufficient correspondences between samples. Even if for a data set with very little correspondences a joint model could be obtained by using a very large number of components, it would hard to gain any insights from such a model. In such a case an entirely computational pipeline without the cell population identification step would be preferred.

BayesFlow can be computationally intensive if many runs are needed to pass the quality control. For these cases it would be desirable to complement BayesFlow e.g. with initialization methods that would allow passing the quality control more often, so that few runs in BayesFlow would be needed. Fast initialization methods and early quality checks aiming at this would therefore be of interest for the community and is something that we propose for further study.

## Conclusions

In this paper we have presented a new Bayesian hierarchical model designed for joint cell population identification in many flow cytometry samples. The model captures the variability in shapes and locations of the populations between the samples and we have demonstrated its use in an exploratory as well as in a partly informed setting with some prior information. We showed that for synthetic datasets generated from the model, the parameters were recovered with high accuracy through a MCMC sampling scheme. The model was then applied to a real flow cytometry data set where a manual gating was available, and it was shown to have very high accordance with manual gating as compared to other automated gating methods, while at the same time the gating was more consistent across samples than either the manual gating or other automated gating methods. When applied to another flow cytometry data set with technical replicates of blood from healthy donors, BayesFlow gave a parsimonious representation of the data, which enables visualization and monitoring of its parameters. The obtained cell populations had clear expression patterns as opposed to the clusters obtained by ASPIRE and HDPGMM, where for example CD4+CD8- T-cells where in the same cluster as CD4+CD8+ T-cells. The population sizes obtained by BayesFlow and HDPGMM respectively had lower intra-donor variation compared to inter-donor variation than what was obtained from ASPIRE.

Many approaches of automated gating of multiple flow cytometry samples in parallel have been aimed at finding features of the data so that either samples can be classified into groups, e.g. cancer or normal, or they can be used to predict an outcome such as expected time to progression of disease. Features are often designed based on characteristics of cell populations, but usually not so much attention has been given to ensure that they represent actual cell populations. BayesFlow takes the opposite approach and gives a representation of the data according to cell populations, with the same cell populations across the entire set of samples (except when some populations only occurs in a subset of the samples). The advantages to this approach are among others that the result is directly biologically interpretable and that a rich output is given which can be explored in many different ways which are familiar to someone who is used to manual gating. In this way we can join the objectivity and ability to work in high dimensions and with many samples of automated gating with the flexibility in interpretation of manual gating.

## Additional files

Additional file 1 **Supplementary material.** The supplementary material contains the posterior in BayesFlow, the MCMC sampling scheme, additional details on the merging of components, information about the data generation, priors and initialization for the synthetic data example; parameters used for ASPIRE, additional details on healthyFlowData, the priors and the initialization procedure used when studying this data set and further results pertaining to the real flow cytometry data set, including fitting Gaussian mixture models to individual samples of healthyFlowData with the EM algorithm and scatter plots of GvHD for ASPIRE, HDPGMM and BayesFlow run 1. (PDF 7505 kb)

Additional file 2 **Data generation files.** A Python script for generating the large synthetic dataset, along with means, covariances and weights needed for this. (ZIP 10kb)
