# Erratum to: BayesFlow: latent modeling of flow cytometry cell populations

**DOI:** 10.1186/s12859-016-0973-1

**Published:** 2016-03-31

**Authors:** Kerstin Johnsson, Jonas Wallin, Magnus Fontes

**Affiliations:** Centre for Mathematical Sciences, Lund University, Box 118, S-221 00 Lund, Sweden; Mathematical Sciences, Chalmers and University of Gothenburg, S-412 58 Gothenburg, Sweden; International Group for Data Analysis, Institut Pasteur, 25 Rue du Docteur Roux, 75015 Paris, France

Unfortunately, the original version of this article [1] contained an error whereby the figures are completely out of order. The correct order of figures is as below. For example, Figure 1 should be the first figure, Figure 6 should be the second figure, Figure 2 should be the third figure and so forth as listed below. This has been corrected in the original article.

Fig. 1

Fig. 6

Fig. 2

Fig. 3

Fig. 7

Fig. 8

Fig. 9

Fig. 10

Fig. 11

Fig. 12

Fig. 13

Fig. 4

Fig. 5

Fig. 14
