# Supplementary material for: BayesFlow: latent modeling of flow cytometry cell populations
Source: BMC Bioinformatics. 2016 Jan 12;17:25. doi: 10.1186/s12859-015-0862-z (PMC4709953; doi:10.1186/s12859-015-0862-z)
Supplement: Supplementary file 1 — Supplementary material. The supplementary material contains the posterior in BayesFlow, the MCMC sampling scheme, additional details on the merging of components, information about the data generation, priors and initialization for the synthetic data example; parameters used for ASPIRE, additional details on healthyFlowData, the priors and the initialization procedure used when studying this data set and further results pertaining to the real flow cytometry data set, including fitting Gaussian mixture models to individual samples of healthyFlowData with the EM algorithm and scatter plots of GvHD for ASPIRE, HDPGMM and BayesFlow run 1. (PDF 7505 kb) [file 12859_2015_862_MOESM1_ESM.pdf]

BayesFlow: Latent modeling of flow cytometry cell populations — Supplemental Material

Kerstin Johnsson*[1], Jonas Wallin[2], and Magnus Fontes[1,3]

[1]Mathematical Sciences, Chalmers and University of Gothenburg
[2]Centre for Mathematical Sciences, Lund University
[3]International Group for Data Analysis, Institut Pasteur, Paris

November 20, 2015

*johnsson@maths.lth.se; Corresponding author

A Posterior

The posterior distribution given the model (1), (2), the priors (3) and data $\mathbf{Y}$ is

$$\begin{aligned}
&\pi(\boldsymbol{\Theta}|\mathbf{Y}, \mathbf{x}) \\
&\propto \left(\prod_{j=1}^{J} \prod_{i=1}^{n_j} |\boldsymbol{\Sigma}_{j\mathbf{x}_{ij}}|^{-1/2} \exp\left(-\frac{1}{2} (\mathbf{Y}_{ij} - \boldsymbol{\mu}_{j\mathbf{x}_{ij}})^\top \boldsymbol{\Sigma}_{j\mathbf{x}_{ij}}^{-1} (\mathbf{Y}_{ij} - \boldsymbol{\mu}_{j\mathbf{x}_{ij}}) \right) \pi_{j\mathbf{x}_{ij}} \right) \cdot \\
&\left(\prod_{j=1}^{J} \prod_{k=1}^{K} \pi_{jk}^{a_{jk}} |\boldsymbol{\Sigma}_{\theta_k}|^{-\frac{1}{2}} \exp\left(-\frac{1}{2} (\boldsymbol{\mu}_{jk} - \boldsymbol{\theta}_k)^\top \boldsymbol{\Sigma}_{\theta_k}^{-1} (\boldsymbol{\mu}_{jk} - \boldsymbol{\theta}_k) \right) \right. \\
&\qquad \left. \frac{|\boldsymbol{\Sigma}_{jk}|^{-\frac{\nu_k+d+1}{2}} |\boldsymbol{\Psi}_k|^{\frac{\nu_k}{2}}}{2^{\frac{\nu_k d}{2}} \Gamma_d(\frac{\nu_k}{2})} \exp\left(-\mathrm{tr}(\boldsymbol{\Psi}_k \boldsymbol{\Sigma}_{jk}^{-1})/2 \right) \right) \cdot \\
&\left(\prod_{k=1}^{K} \exp\left(-\frac{1}{2} (\boldsymbol{\theta}_k - \mathbf{t}_k)^\top \mathbf{S}_k^{-1} (\boldsymbol{\theta}_k - \mathbf{t}_k) \right) |\boldsymbol{\Psi}_k|^{\frac{n_{\Psi_k}-d-1}{2}} \exp\left(-\mathrm{tr}(\mathbf{H}_k^{-1} \boldsymbol{\Psi}_k)/2 \right) \right. \\
&\qquad \left. |\boldsymbol{\Sigma}_{\theta_k}|^{-\frac{n_{\theta_k}}{2}} \exp\left(-\mathrm{tr}(\mathbf{Q}_k \boldsymbol{\Sigma}_{\theta_k}^{-1})/2 \right) \exp(-\lambda_k \nu_k) \right). \quad (1)
\end{aligned}$$

B Sampling from the posterior distribution

We use a Markov Chain Monte Carlo (MCMC) algorithm to generate samples from the posterior distribution of the parameters [1]. In each iteration we draw a value of each of the parameters $\boldsymbol{\Theta}$ and of $\mathbf{x}$. The backbone of our algorithm is a Gibbs sampler, but we need a Metropolis-Hastings step to sample ν_k. We also use Metropolis-Hastings steps to enable label-switching—which improves the mixing of the Gibbs sampler—and to turn on and off mixture components in the extended model with absent clusters.

In a Gibbs sampler samples from the full posterior distribution is obtained by successively sampling from the conditional posterior distributions of each of the variables given all other variables. First we sample the component assigment variables, $\mathbf{x}$, fixing all other parameters. The posterior from which we sample is a multinomial distribution with

$$\pi(x_{ij} = k|\ldots) \propto \frac{N(\mathbf{Y}_{ij}; \boldsymbol{\mu}_{jk}, \boldsymbol{\Sigma}_{jk})\pi_{jk}}{\sum_{h=0}^{K} N(\mathbf{Y}_{ij}; \boldsymbol{\mu}_{jh}, \boldsymbol{\Sigma}_{jh})\pi_{jh}},$$

where '...' denotes conditioning on all parameter except the one of interest.

Let n_{jk} denote the number of i such that $x_{ij} = k$ and let $\mathbf{Y}_{.jk}$ denote the vector joining all $\mathbf{Y}_{ij}$ such that $x_{ij} = k$. The following Gibbs steps are derived from the posterior distribution (1)

$$\begin{aligned}
\boldsymbol{\pi}_j|\ldots &\sim D(a+n_{j1},\ldots,a+n_{jK}), \qquad (2)\\
\boldsymbol{\Sigma}_{jk}|\ldots &\sim IW(\boldsymbol{\Psi}_k + \sum_{i=1}^{n_{jk}}(\mathbf{Y}_{ijk}-\boldsymbol{\mu}_{jk})(\mathbf{Y}_{ijk}-\boldsymbol{\mu}_{jk})^\top, n_{jk}+\nu_k),\\
\boldsymbol{\mu}_{jk}|\ldots &\sim N_C(\boldsymbol{\Sigma}_{\theta_k}^{-1}\boldsymbol{\theta}_k + \boldsymbol{\Sigma}_{jk}^{-1}\sum_{i=1}^{n_{jk}}\mathbf{Y}_{ijk}, \boldsymbol{\Sigma}_{\theta_k}^{-1}+n_{jk}\boldsymbol{\Sigma}_{jk}^{-1}),\\
\boldsymbol{\Sigma}_{\theta_k}|\ldots &\sim IW(\mathbf{Q}_k + \sum_{j=1}^{J}(\boldsymbol{\mu}_{jk}-\boldsymbol{\theta}_k)(\boldsymbol{\mu}_{jk}-\boldsymbol{\theta}_k)^\top, J+n_{\theta_k}),\\
\boldsymbol{\Psi}_k|\ldots &\sim W\left(\left(\mathbf{H}_k^{-1}+\sum_{j=1}^{J}\boldsymbol{\Sigma}_{jk}^{-1}\right)^{-1}, n_{\Psi_k}+J\nu_k\right),\\
\boldsymbol{\theta}_k|\ldots &\sim N_C(\mathbf{S}_k^{-1}\mathbf{t}_k + \boldsymbol{\Sigma}_{\theta_k}^{-1}\sum_{j=1}^{J}\boldsymbol{\mu}_{jk}, \mathbf{S}_k^{-1}+J\boldsymbol{\Sigma}_{\theta_k}^{-1}).
\end{aligned}$$

Here N_C denotes the canonical parameterization of the normal distribution, which means that if $\mathbf{x} \sim N_C(\mathbf{b},\mathbf{Q})$, then $\pi(\mathbf{x}) \propto \exp\left(-\frac{1}{2}\mathbf{x}^\top\mathbf{Q}\mathbf{x}+\mathbf{b}^\top\mathbf{x}\right)$.

To handle the non-standard conditional distribution of ν_k, we utilize a Metropolis-Hastings (MH) algorithm. A proposal ν_k^* is generated by sampling $\nu_k^* \sim \nu_k + Z$, where Z is uniformly distributed on $\{-r, -r+1, \ldots, r\}$ for some $r \in \mathbb{N}^+$. Hence the transition density $q(\nu_k,\nu_k^*) = q(\nu_k|\nu_k^*)$ is constant on its support. The proposed ν_k^* is accepted with probability

$$\alpha(\nu_k,\nu_k^*) = \min\left(1, \frac{\pi(\nu_k^*)q(\nu_k^*,\nu)}{\pi(\nu_k)q(\nu,\nu_k^*)}\right) = \min\left(1, \frac{\pi(\nu_k^*)}{\pi(\nu_k)}\right),$$

where $\pi(\nu_k)$ denotes the posterior distribution of ν_k given all other parameters and data. Using (1) we get that

$$\alpha(\nu_k,\nu_k^*) = \min\left(1, \prod_{j=1}^{J}\frac{\Gamma_d(\frac{\nu_k}{2})}{\Gamma_d(\frac{\nu_k^*}{2})}\left(2^d|\boldsymbol{\Sigma}_{jk}||\boldsymbol{\Psi}_k|^{-1}\right)^{\frac{\nu_k-\nu_k^*}{2}}\exp\left(\lambda_k(\nu_k^*-\nu_k)\right)\right).$$

If ν_k^* is accepted it will be the new sample, otherwise the new sample will be ν_k. The parameter r is updated adaptively to get a desired acceptance rate of 0.3, according to an algorithm by Roberts and Rosenthal [2].

B.1 Label switching

An issue that frequently occurs, especially with poor starting values, is that a cluster $\{\boldsymbol{\mu}_{jk_1}, \boldsymbol{\Sigma}_{jk_1}, \pi_{jk_1}\}$ is incorrectly assigned to the latent cluster k_1 when it clearly should belong to k_2. When the number cells is large the first row of (1) will dominate the posterior so that $\{\boldsymbol{\mu}_{jk_1}, \boldsymbol{\Sigma}_{jk_1}, \pi_{jk_1}\}$ or $\{\boldsymbol{\mu}_{jk_2}, \boldsymbol{\Sigma}_{jk_2}, \pi_{jk_2}\}$ does not change much at all in the updating step and thus in practice the clusters will never move close enough to each other in order to switch locations.

To remedy this issue, we introduce an extra MH step where labels can be switched between clusters in each sample j in each iteration. The proposed MH algorithm has a symmetric transition kernel, where two labels k_1 and k_2 are sampled from $\{1, \ldots, K\}$ with equal probability. The proposed switch is accepted with probability

$$\alpha(k_1, k_2) = \min\left(1, \frac{\pi(\mu_{jk_2}|\boldsymbol{\theta}_{k_1}, \boldsymbol{\Sigma}_{\theta_{k_1}})\pi(\mu_{jk_1}|\boldsymbol{\theta}_{k_2}, \boldsymbol{\Sigma}_{\theta_{k_2}})}{\pi(\mu_{jk_1}|\boldsymbol{\theta}_{k_1}, \boldsymbol{\Sigma}_{\theta_{k_1}})\pi(\mu_{jk_2}|\boldsymbol{\theta}_{k_2}, \boldsymbol{\Sigma}_{\theta_{k_2}})} \frac{\pi(\boldsymbol{\Sigma}_{jk_1}|\boldsymbol{\Psi}_{k_2}, \nu_{k_2})\pi(\boldsymbol{\Sigma}_{jk_2}|\boldsymbol{\Psi}_{k_1}, \nu_{k_1})}{\pi(\boldsymbol{\Sigma}_{jk_1}|\boldsymbol{\Psi}_{k_1}, \nu_{k_1})\pi(\boldsymbol{\Sigma}_{jk_2}|\boldsymbol{\Psi}_{k_2}, \nu_{k_2})}\right). \quad (3)$$

B.2 Cluster activation and deactivation

In the extended model where components can be absent in some samples we use a reversible jump MH-algorithm [3] to enable changes to the dimension of the model. We use the indicator variable $\mathbf{Z}_j$ to keep track of which components that are active; $Z_{jk} = 1$ if component k is active in sample j and $Z_{jk} = 0$ otherwise.

Activation or deactivation is proposed as the last step of each iteration of the MCMC algorithm. Throughout the activation/deactivation step the component assignment variables x_{ij} are integrated out of the posterior.

A deactivation of an active component is proposed with probability p_d and an activation of a component that is not active is proposed with probability p_a. The component that is proposed to be deactivated/activated is chosen randomly among the clusters that are active or not active respectively with

equal probability. The probability of proposing to deactivate component k in sample j is

$$q(Z_{jk} = 1 \to 0) = \frac{p_d}{\sum_{l=1}^{K} Z_{jl}}.$$

The probability of proposing to activate component k in sample j is

$$q(Z_{jk} = 0 \to 1) = \frac{p_a}{K - \sum_{l=1}^{K} Z_{jl}}.$$

If an activation step is proposed it is necessary to generate parameters for the new component; they are obtained in the following way:

$$\begin{aligned} \pi_{jk}^* &\sim \text{Beta}(\alpha, \beta), \\ \boldsymbol{\mu}_{jk}^* &\sim N(\boldsymbol{\theta}_k, \boldsymbol{\Sigma}_{\theta_k}), \\ \boldsymbol{\Sigma}_{jk}^* &\sim IW(\boldsymbol{\Psi}_k, \nu_k). \end{aligned}$$

Here α and β is chosen so that the probability π_{jk}^* is typically close to zero. The transition density $q_{k_j}(\boldsymbol{\mu}_{jk}^*, \boldsymbol{\Sigma}_{jk}^*, \pi_{jk}^*)$ is the joint density of these new parameters when they are sampled as above. For the remaining components we keep the mean and covariance parameters, $\boldsymbol{\mu}_{jl}^* = \boldsymbol{\mu}_{jl}$ and $\boldsymbol{\Sigma}_{jl}^* = \boldsymbol{\Sigma}_{jl}$ for $l \neq k$, but the probabilities $\boldsymbol{\pi}_j$ have to be modified. In the reversible jump algorithm this is done in a dimension matching transform. When activating a cluster we set $\pi_{jl}^* = (1 - \pi_{jk}^*)\pi_{jl}$ for $l \neq k$ in the transform and when deactivating a cluster we set $\pi_{jl}^* = \pi_{jl}/(1 - \pi_{jk})$ for $l \neq k$.

In order to make the Markov chain reversible it is necessary to add the Jacobian of the variable change in the dimension matching transform as a factor in the acceptance probability. Let $\boldsymbol{\Theta}^*$ denote the set of parameters in the proposed model and let $\boldsymbol{\Theta}$ denote the set of current parameters. In an activation step we get [4]

$$\left| \frac{\partial(\boldsymbol{\Theta}^*)}{\partial\left(\boldsymbol{\Theta}, \pi_{jk}^*, \boldsymbol{\mu}_{jk}^*, \boldsymbol{\Sigma}_{jk}^*\right)} \right| = (1 - \pi_{jk}^*)^{\sum_{l=1}^{K} Z_{jl}},$$

and in a deactivation step the Jacobian is the inverse.

We are now ready to define the acceptance probability for a proposed $\boldsymbol{\Theta}^*$ which implies activation of component k in sample j. The acceptance

probability equals

$$\alpha\left(\boldsymbol{\Theta},\, \boldsymbol{\Theta}^{*}\right) = \min\left\{1, \frac{\pi(\boldsymbol{\Theta}^{*}|\mathbf{Y})q(Z_{jk}=1\to 0)}{\pi(\boldsymbol{\Theta}|\mathbf{Y})q_{k_j}(\boldsymbol{\mu}^{*}_{jk}, \boldsymbol{\Sigma}^{*}_{jk}, \pi^{*}_{jk})q(Z^{*}_{jk}=0\to 1)} \left|\frac{\partial(\boldsymbol{\Theta}^{*})}{\partial\left(\boldsymbol{\Theta}, \pi^{*}_{jk}, \boldsymbol{\mu}^{*}_{jk}, \boldsymbol{\Sigma}^{*}_{jk}\right)}\right|\right\}, \quad (4)$$

where $\pi(\boldsymbol{\Theta}^{*}|\mathbf{Y})$ is the posterior distribution (1) with x_{ij} integrated out. This can be written as

$$\alpha\left(\boldsymbol{\Theta},\, \boldsymbol{\Theta}^{*}\right) = \min\left\{1, \frac{\prod_{i=1}^{n_j}\sum_{l=1}^{K} Z^{*}_{jl}\pi^{*}_{jl}N(\mathbf{Y}_{ij}; \boldsymbol{\mu}^{*}_{jl}, \boldsymbol{\Sigma}^{*}_{jl})}{\prod_{i=1}^{n_j}\sum_{l=1}^{K} Z_{jl}\pi_{jl}N(\mathbf{Y}_{ij}; \boldsymbol{\mu}_{jl}, \boldsymbol{\Sigma}_{jl})} \cdot \frac{D(\boldsymbol{\pi}^{*}_{j}; \mathbf{a})\exp(-c_s)}{\mathrm{Beta}(\pi^{*}_{jk}; \alpha, \beta)D(\mathbf{p}_j; \mathbf{a})} \frac{\frac{p_d}{\sum_{l=1}^{K} Z_{jl}}}{\frac{p_b}{K-\sum_{l=1}^{K} Z_{jl}}}(1-\pi^{*}_{jk})^{\sum_{l=1}^{K} Z_{jl}}\right\}.$$

The acceptance probability for a deactivation step is obtained from the same expression but with inverse ratio.

When we extend the model and introduce $\mathbf{Z}_j$ the posterior changes so that the sampling of the other variables has to be modified. As an example the conditional distribution of $\boldsymbol{\Psi}_k$ changes to

$$W\left(\left(\mathbf{H}_k + \sum_{h=1}^{J} Z_{hk}\boldsymbol{\Sigma}^{-1}_{jk}\right)^{-1},\ \nu^{*} + \nu_k\sum_{h=1}^{J} Z_{hk}\right).$$

We do not display all the changes since they are notationally complicated but otherwise straightforward, except for the label switching step. Suppose we propose to change k_1 to k_2 where k_1 is an inactive cluster. Then the acceptance probability (3) changes to

$$\alpha(k_1, k_2) = \min\left(1, \frac{\pi(\boldsymbol{\mu}_{jk_2}|\boldsymbol{\theta}_{k_1}, \boldsymbol{\Sigma}_{\theta_{k_1}})\pi(\boldsymbol{\Sigma}_{jk_2}|\boldsymbol{\Psi}_{k_1}, \nu_{k_1})}{\pi(\boldsymbol{\mu}_{jk_2}|\boldsymbol{\theta}_{k_2}, \boldsymbol{\Sigma}_{\mu_{k_2}})\pi(\boldsymbol{\Sigma}_{jk_2}|\boldsymbol{\Psi}_{k_2}, \nu_{k_2})}\right). \quad (5)$$

C Merging latent clusters

The merging of latent clusters is done in a hierarchical fashion. In each step we have a number of latent super clusters comprising of one or more latent

clusters. The corresponding super components in each sample are mixtures of Gaussians, a representation which is hard to work with. It is useful to instead use the data perspective, i.e. to consider the soft clustering of the data induced by the GMM of each sample.

For each sample we define super cluster k from the probabilities for each of the data points in that sample to belong to any of the components linked to the latent super cluster k. We denote cluster k in sample j by $\Gamma_{k,j} = (\mathbf{Y}_{ij}, w_{ijk})_{i=1}^{n_j}$, where w_{ijk} is the probability that $\mathbf{Y}_{ij}$ belongs to super cluster k. The parameter w_{ijk} can be estimated from the sampling of x_{ij}.

To determine candidates for the subsequent merger, Bhattacharyya distance is computed between all pairs of current clusters in each sample. To do this we approximate each $\Gamma_{k,j}$ with a Gaussian distribution with parameters

$$\boldsymbol{\mu}^{(kj)} = \sum_{i=1}^{n_j} w_{ijk}\mathbf{Y}_{ij}, \qquad \boldsymbol{\Sigma}^{(kj)} = \sum_{i=1}^{n_j} w_{ijk}(\mathbf{Y}_{ij} - \boldsymbol{\mu}^{(kj)})(\mathbf{Y}_{ij} - \boldsymbol{\mu}^{(kj)})^\top$$

and use formula (4), so

$$d_{\text{bhat}}(\Gamma_{k,j}, \Gamma_{l,j}) = 1/8 \cdot (\boldsymbol{\mu}^{(kj)} - \boldsymbol{\mu}^{(lj)})^\top \bar{\boldsymbol{\Sigma}}^{-1} (\boldsymbol{\mu}^{(kj)} - \boldsymbol{\mu}^{(lj)}) + 1/2 \cdot \log\left(|\bar{\boldsymbol{\Sigma}}| / \sqrt{|\boldsymbol{\Sigma}^{(kj)}||\boldsymbol{\Sigma}^{(lj)}|}\right),$$

where $\bar{\boldsymbol{\Sigma}} = (\boldsymbol{\Sigma}^{(kj)} + \boldsymbol{\Sigma}^{(lj)})/2$. The candidates for the subsequent merger are the pair of clusters (k, l)—which among those pairs who have not previously been evaluated for merging—has highest minimal value of $\exp(-d_{\text{bhat}}(\Gamma_{kj}, \Gamma_{lj}))$ across samples j. It is natural to consider $\exp(-d_{\text{bhat}})$ instead of d_{bhat} when comparing Bhattacharyya distances since $\exp(-d_{\text{bhat}})$ is an upper bound of the misclassification probability between the components [5].

If $\min_j(\exp(-d_{\text{bhat}}(\Gamma_{kj}, \Gamma_{lj}))) > h_1$ latent clusters k and l are immediately merged. On the other hand, if $h_1 > \min_j(\exp(-d_{\text{bhat}}(\Gamma_{kj}, \Gamma_{lj}))) > h_2$, they are merged only if the resulting cluster does not have sufficient evidence of being multimodal. Finally, if $\min_j(\exp(-d_{\text{bhat}}(\Gamma_{kj}, \Gamma_{lj}))) < h_2$ they are not merged and the procedure is stopped.

To evaluate multimodality of potential mergers we apply Hartigan's dip test of unimodality [6] to the projection of the merged cluster onto the coordinate axes which have 1-dimensional Bhattacharyya overlap below a threshold $h^{(1)}$ and to the projection onto Fisher's discriminant coordinate separating the two clusters, namely $u = (\boldsymbol{\Sigma}^{(kj)} + \boldsymbol{\Sigma}^{lj})^{-1}(\boldsymbol{\mu}^{(kj)} - \boldsymbol{\mu}^{(lj)})$ [7]. Hartigan's

dip statistic is computed from the empirical distribution function, which can readily be computed for these soft clusters from $(\mathbf{Y}_{ij}, w_{ijk})_{i=1}^{n_j}$. If for any of the projections in any of the samples where the total weight of the cluster $\sum_i^{n_j} w_{ijk}$ is at least 10, we get a p-value below the threshold h_d we do not merge.

To determine the thresholds h_1, h_2 and h_d we use results from two experiments performed by Hennig [8]. Synthetic data were generated from distributions which naturally represent a single cluster and a number of Gaussian components were fitted to the data. For different criteria, threshold values for merging the components to one cluster in 95% of the cases, were then reported. The experiments were performed over a range of different dimensions and number of data points. To determine h_1, h_2 and h_d, we consider only results for distributions of dimension two to five and for at least 100 and at most 500 points, since for most of the flow cytometry samples in the data sets studied in Section a small cluster containing 1% of the data points would have about 100–200 data points.

In the first experiment two components were fitted to data generated from a unimodal mixture of two Gaussian distributions with the property that if the means were further apart the density would be bimodal. In the second experiment six Gaussian components were fitted to data generated from uniform distributions on hypercubes. The merging of the six components were made in a hierarchical procedure similar to ours.

When Bhattacharyya distance was used as merging criterion the threshold for $\exp(-d_{\text{bhat}})$ varied between 0.40 and 0.53 for the relevant 2- and 5-dimensional data sets in the first experiment. For the second experiment we considered four combinations of dimension and number of data points and for these the thresholds were 0.12, 0.17, 0.01 and 0.11 respectively. This lead us to use $h_1 = 0.47$ as the soft threshold and $h_2 = 0.08$ as the soft threshold.

Hartigan's dip test was also evaluated as a criterion for merging, but only the first of the experiments is relevant for our use of it, since we only use the dip test to evaluate proposed mergers and not select candidates for merging. Only projections onto Fisher's discriminant coordinate were considered in the experiment. The threshold for the p-value varied between 0.15 and 0.41, so we chose $h_d = 0.28$. It should be noted that this cannot be translated into a significance level since the tests are done in a data-dependent way.

The threshold $h^{(1)}$ was set based on the results in the first experiment for one-dimensional data sets. For data sets with 50 data points, the threshold for $\exp(-d_{\text{bhat}})$ was 0.201, for data sets with 200 points it was 0.39 and for

data sets with 500 data points it was 0.49. Therefore we let $h^{(1)}$ be dependent on the weight of the cluster in the following way:

$$h^{(1)}(w) = \begin{cases} 0.201 & \text{if} \quad w \leq 50 \\ 0.390 & \text{if} \quad 50 < w \leq 200 \\ 0.490 & \text{if} \quad w > 200. \end{cases}$$

D Simulation study

D.1 Data generation

In this section the method for generating the small synthetic dataset is presented. The Additional file `article_simulatedata.py` contains the method for generating the large synthetic dataset. The four latent means are

$$\boldsymbol{\theta}_1 = [0, 0, 0],\ \boldsymbol{\theta}_2 = [0, -2, 1],\ \boldsymbol{\theta}_3 = [1, 2, 0],\ \boldsymbol{\theta}_4 = [-2, 2, 1.5].$$

Each $\boldsymbol{\mu}_{jk}$ in the simulation is generated by

$$\begin{aligned} \boldsymbol{\mu}_{jk} &= \boldsymbol{\theta}_k + \mathbf{Z}_{jk},\ k = 1, 2, 3, 4 \\ \mathbf{Z}_{jk} &\sim\ N(\mathbf{0}, \boldsymbol{\Sigma}_{\mu_k}), \end{aligned}$$

where

$$\boldsymbol{\Sigma}_{\mu_1} = \begin{bmatrix} 1.27 & 0.25 & 0 \\ 0.25 & 0.27 & -0.001 \\ 0 & -0.001 & 0.001 \end{bmatrix}, \qquad \boldsymbol{\Sigma}_{\mu_2} = \begin{bmatrix} 0.06 & 0.04 & -0.03 \\ 0.04 & 0.05 & 0 \\ -0.03 & 0. & 0.09 \end{bmatrix},$$

$$\boldsymbol{\Sigma}_{\mu_3} = \begin{bmatrix} 0.44 & 0.08 & 0.08 \\ 0.08 & 0.16 & 0 \\ 0.08 & 0 & 0.16 \end{bmatrix}, \qquad \boldsymbol{\Sigma}_{\mu_4} = 0.01\mathbf{I}.$$

The covariance matrices are generated through

$$\boldsymbol{\Sigma}_{jk} \sim IW((\nu_k - 3)\boldsymbol{\Psi}_k, \nu_k),\ k = 1, 2, 3, 4,$$

where

$$\boldsymbol{\Psi}_1 = 0.1\mathbf{I}, \qquad \boldsymbol{\Psi}_2 = 0.1 \begin{bmatrix} 2.0 & 0.5 & 0 \\ 0.5 & 2.0 & 0.5 \\ 0 & 0.5 & 2.0 \end{bmatrix},$$

$$\boldsymbol{\Psi}_3 = 0.1 \begin{bmatrix} 2.0 & -0.5 & 1.0 \\ -0.5 & 2.0 & -0.5 \\ 1.0 & -0.5 & 2.0 \end{bmatrix}, \qquad \boldsymbol{\Psi}_4 = 0.1 \begin{bmatrix} 1.0 & 0.3 & 0.3 \\ 0.3 & 1.0 & 0.3 \\ 0.3 & 0.3 & 1.0 \end{bmatrix},$$

and $\nu_k = 100$ for all k. Finally, $\boldsymbol{\pi}_j = [0.49, 0.3, 0.2, 0.01]$ if all clusters are present. If one or two clusters are not present the ratio of the probabilities for the present clusters remains the same.

D.2 Priors

The priors are set to represent non informative priors; the priors are set equal for all classes. The exact values are:

$$\begin{aligned}\mathbf{S}_k &= 10^6\mathbf{I}_d, \mathbf{t}_k = \mathbf{0},\\ \mathbf{H}_k &= 10^{-6}\mathbf{I}_d, n_{\psi_k} = d,\\ \mathbf{Q}_k &= 10^{-6}\mathbf{I}_d, n_{\theta_k} = d,\\ l_k &= 0.01,\end{aligned}$$

for $k = 1, 2, 3, K$ with $K = 4$ for the small dataset and $K = 11$ for the large dataset. For the small dataset the outlier component was not used for inference.

D.3 Initialization for small dataset

Before running the MCMC sampler to get samples from the posterior distribution, we utilize the following initialization to get suitable initial parameter values. First we set all mean parameters $\boldsymbol{\mu}_{jk}$ and $\boldsymbol{\theta}_k$ to $\mathbf{0}$ and all covariance and precision matrices $\boldsymbol{\Sigma}_{jk}$, $\boldsymbol{\Sigma}_{\theta_k}$ and $\boldsymbol{\Psi}_k$ to $\mathbf{I}$. Then after letting the MCMC sampler run for 5000 iterations, without the option of turning off components, we link all the components across samples through the following procedure:

1. The first sample is left unchanged.

2. For the second sample the components are first sorted by $\boldsymbol{\pi}_2$, so we get ordered components $(\boldsymbol{\mu}_{2(i)}, \boldsymbol{\Sigma}_{2(i)}, \pi_{2(i)})$ for $i = 1, 2, 3, 4$, where $\pi_{2(1)} \geq \pi_{2(2)} \geq \pi_{2(3)} \geq \pi_{2(4)}$. Then the first component $(\boldsymbol{\mu}_{2(1)}, \boldsymbol{\Sigma}_{2(1)}, \pi_{2(1)})$ is matched to the component k whose mean $\boldsymbol{\mu}_{1k}$ is closest to $\boldsymbol{\mu}_{2(1)}$. If for example we have that $\boldsymbol{\mu}_{13}$ is closest to $\boldsymbol{\mu}_{2(1)}$ we set $(\boldsymbol{\mu}_{23}, \boldsymbol{\Sigma}_{23}, \pi_{23}) = (\boldsymbol{\mu}_{2(1)}, \boldsymbol{\Sigma}_{2(1)}, \pi_{2(1)})$. This is repeated for $(\boldsymbol{\mu}_{2(i)}, \boldsymbol{\Sigma}_{2(i)}, \pi_{2(i)})$, $i = 2, 3, 4$, but indices which have already been assigned to components are excluded from consideration.

3. For the remaining samples we proceed as for the second sample, with the exception that the matching of $\boldsymbol{\mu}_{j(k)}$ is now done to the average of the $j-1$ previously matched clusters means, namely $(j-1)^{-1}\sum_{l=1}^{j-1}\boldsymbol{\mu}_{lk}$ for $k = 1, 2, 3, 4$.

D.4 Initialization for large dataset

Before starting the actual MCMC sampler, we run an initialization scheme that is designed to make the sampler jump out of local maxima of the likelihood. The method we use does not give a reversible Markov chain and thus cannot be part of the actual MCMC run. We do the following steps about ten times for each GMM without updating the latent parameters:

1. Sample $\mathbf{x}, \boldsymbol{\mu}, \boldsymbol{\Sigma}, \boldsymbol{\pi}_j$ using the regular Gibbs sampler for ten iterations.

2. Calculate the likelihood for the current parameters $\boldsymbol{\mu}, \boldsymbol{\Sigma}, \boldsymbol{\pi}_j$. Randomly select a cluster $(\boldsymbol{\mu}_k, \boldsymbol{\Sigma}_k, \pi_{jk})$ and then select a dimension d_1 at random. Remove the cluster $(\boldsymbol{\mu}_k, \boldsymbol{\Sigma}_k, \pi_{jk})$ and the cluster closest to it in d_1, draw two random points and use them as initial points for two new clusters. Run the Gibbs sampler for ten iterations. If the new parameters has higher likelihood then the old keep the new, otherwise go back to the old.

3. Calculate the likelihood for the current parameters $\boldsymbol{\mu}, \boldsymbol{\Sigma}$. Randomly select a cluster $(\boldsymbol{\mu}_k, \boldsymbol{\Sigma}_k, \pi_k)$, with a probability of choosing cluster k proportional to $\frac{1}{\pi_{jk}}$ so that the smaller the cluster the more likely it is to be chosen. Remove $\boldsymbol{\mu}_k$, draw a random point and use it as $\boldsymbol{\mu}_k$ and set $\boldsymbol{\Sigma}_k$ to the old $\boldsymbol{\Sigma}_k$ times ten. Then run the Gibbs sampler for ten iterations. If the new parameters has higher likelihood then the old keep the new otherwise go back to the old.

The two last steps works quite well for destroying clusters that have been stuck in the wrong shape or removing small clusters that is at the wrong location of the space.

E Flow cytometry data analysis

E.1 Dataset details

E.1.1 healthyFlowData

Here follows a description of the dataset healthyFlowData: how it was obtained and how it was preprocessed before we downloaded it from the R package healthyFlowData.

Antibodies against CD45, CD19, CD3, CD8 and CD4 linked to fluorochromes were used to mark the PBMC and when passed through the flow cytometer the expression of these markers were measured along with front and side scatter. A standard transformation called compensation was used to remove effects of spectral overlap [9]. Following this the data was transformed using the function $\operatorname{asinh}(y/c)$, where c was chosen to minimize Bartlett's statistic, with the purpose to stabilize variance between markers; functions for this transformation are available in the R package flowVS [10]. Measurements corresponding to lymphocytes were selected using front and side scatter by fitting a bivariate normal distribution and filtering based on a likelihood threshold using the norm2Filter function in the flowCore R package (Azad, personal communication). This resulted in between 6172 and 19,554 cell measurements for each sample. Since all lymphocytes are CD45+, only the other four markers were retained.

E.2 Priors

Priors should be set depending on the application, since they specify our tolerance to variation. However, to simplify this process we want to be able to translate prior parameters between data sets with different number of samples, cells and components. To do this, we consider the sampling scheme (2). Looking at the sampling of $\boldsymbol{\theta}_k$, we see that the effect of $\mathbf{S}_k$ decreases proportionally to the number of samples J. Thus we set $\mathbf{S}_k = \mathbf{I} \cdot s_k/J$ for those k for which we want an informative prior on location. Based on the sampling of $\boldsymbol{\mu}_{jk}$ we see that $\boldsymbol{\Sigma}_{\theta_k}$ should be proportional to $1/n_{jk}$, thus from the sampling of $\boldsymbol{\Sigma}_{\theta_k}$, n_{θ_k} should be proportional to n_{jk}. The value n_{jk} can be estimated by n/K, where n is the total number of cells across samples. Furthermore, $\mathbf{Q}_k$ should be proportional to J.

Moving over to shape variation, from the sampling of $\boldsymbol{\Sigma}_{jk}$ we see that $\boldsymbol{\Psi}_k$ should be proportional to n_{jk} and from the sampling of $\boldsymbol{\Psi}$ we see that to

achieve this $n_{\mathbf{\Psi}_k}$ should also be proportional to n_{jk}. Furthermore $\mathbf{H}_k$ should be proportional to $1/J$. In summary,

$$\begin{aligned} n_{\boldsymbol{\theta}_k} &= nt_k \cdot n/K, & n_{\mathbf{\Psi}_k} &= np_k \cdot n/K, \\ \mathbf{Q}_k &= q_k \cdot J, & \mathbf{H}_k &= h_k/J, \\ \mathbf{S}_k &= s_k/J \cdot \mathbf{I}, \end{aligned}$$

where the parameters nt_k, np_k, q_k, h_k and s_k can be reused across data sets of different sizes and with different number of components. Note that $\mathbf{S}_k$ should only be set as above when informative priors on latent locations of clusters are wanted. For the flow cytometry data sets considered in this work we use $nt_k = 0.75$, $np_k = 0.25$, $h_k = 10^3$ and $q_k = 10^{-3}$ for all components k. $\mathbf{S}_k$ was uninformative in most cases and set to 10^6, but for the rare phenotype in the GvHD data set we used $s_k = 0.01^2$.

E.3 Point estimates

During the production iterations of the MCMC sampler we get samples of

$$\mathbf{\Theta}^{(r)} = \left(\boldsymbol{\mu}_{jk}^{(r)}, \mathbf{\Sigma}_{jk}^{(r)}, \boldsymbol{\theta}_k^{(r)}, \mathbf{\Psi}_k^{(r)}, \nu_k^{(r)}, \boldsymbol{\pi}_j^{(r)}\right), \; r = 1, \ldots, R.$$

We use the means of $\boldsymbol{\mu}_{jk}^{(r)}, \mathbf{\Sigma}_{jk}^{(r)}, \boldsymbol{\theta}_k^{(r)}$ and $\mathbf{\Psi}_k^{(r)}/(\nu_k^{(r)} - d - 1)$ to get point estimates of sample component and latent cluster means and covariance matrices; the means of $\boldsymbol{\pi}_j^{(r)}$ are used to get point estimates of the mixing proportions.

E.4 Quality control

E.4.1 Convergence

We assess the convergence of the MCMC sampler in BayesFlow by looking at trace plots for $\boldsymbol{\theta}_k$ and ν_k, where $k \in \{1, \ldots, K\}$. The trace plots for the first accepted run of healthyFlowData and GvHD are shown in Fig. S1 and Fig. S2 respectively.

E.4.2 Unimodality

We want to detect if the distribution of data assigned to a single component or super component is not unimodal, since it indicates that the latent cluster maybe should be divided into two or more components. To do this we use

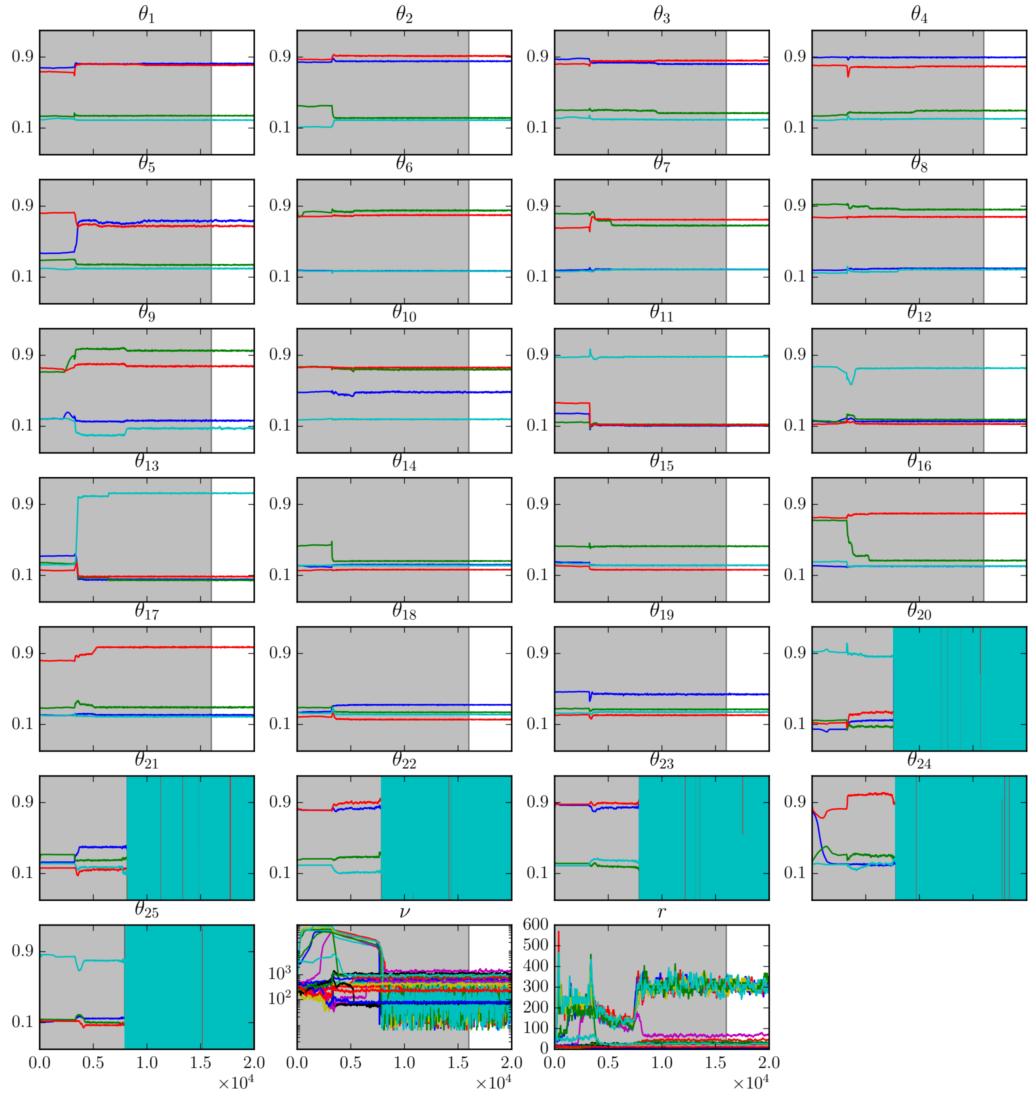

Figure S1: Trace plots of latent means θ_k for $k = 1, \ldots, 25$, ν and MH sampling interval r, for the first accepted BayesFlow run on healthyFlowData. Burn-in iterations are plotted on gray background. As can be seen the clusters 20-25 were turned of during the burn-in iterations.

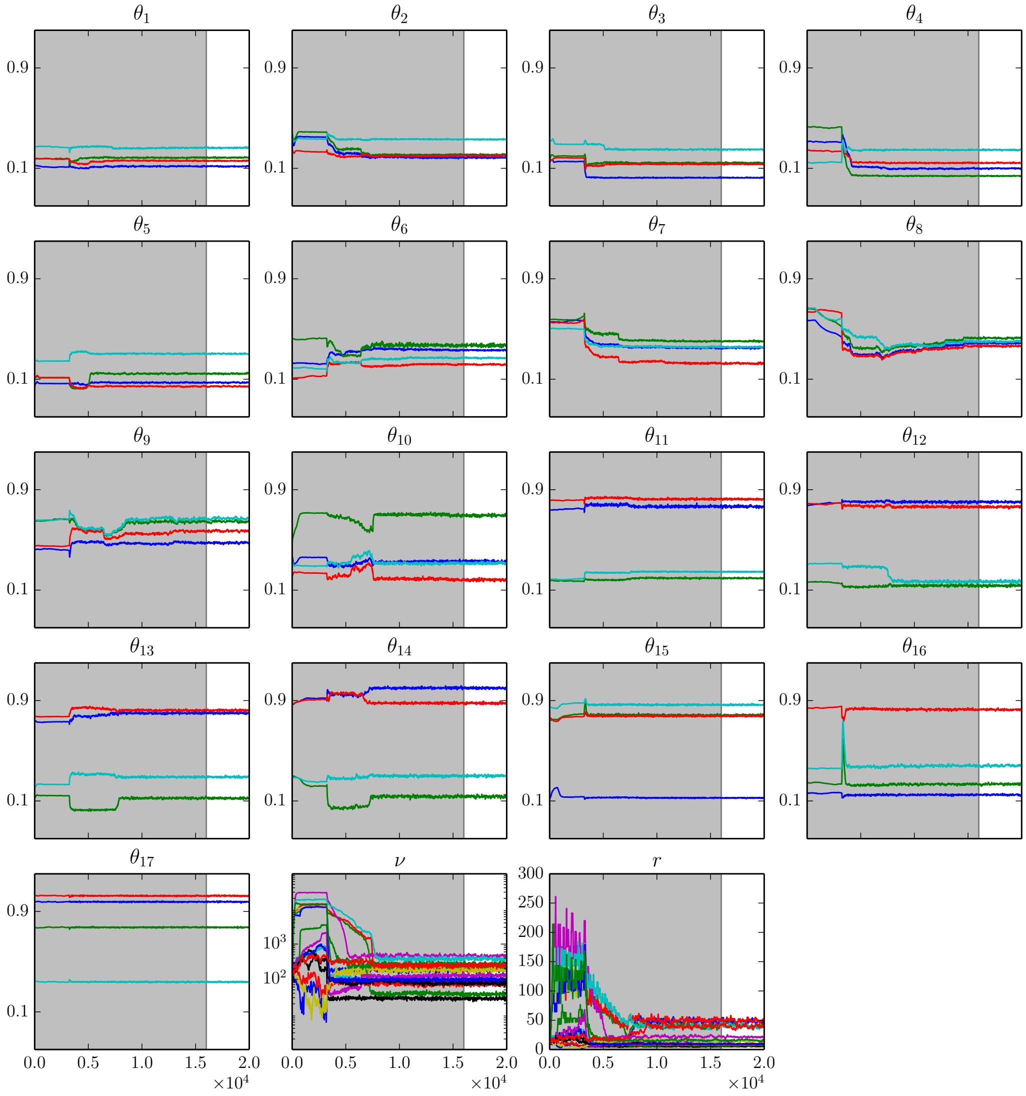

Figure S2: Trace plots of latent means θ_k for $k = 1, \ldots, 25$, ν and MH sampling interval r, for the first accepted BayesFlow run on GvHD. Burn-in iterations are plotted on gray background.

	p_{sw}	p_a=p_d
Burn-in phase 1a	0.1	0
Burn-in phase 1b	0.1	0
Burn-in phase 2a	0.1	0
Burn-in phase 2b	0.1	0.1
Burn-in phase 3	0	0.1
Production phase	0	0.1

Table S1: Simulation parameters for MCMC sampling for real flow cytometry data. During the phase 1a, the prior parameters n_θ and n_Ψ are increased by a factor of 100. After phase 1b, outlying sample components are turned off, i.e. sample components which are closer in Bhattacharyya distance to another latent component than the one to which they are connected.

Hartigan's dip test [6] of unimodality for the one-dimensional marginal distributions. For cluster–dimension combinations which give dip tests below 0.28 (our threshold for merging clusters) we consider histograms of quantiles of the clusters as shown in Fig. S3 (usual histograms are less useful since the clusters are soft). When there are tendencies of bimodality it can be accepted when it seems unlikely that dividing the cluster further would result in a new interesting population. This can for example be the case if this tendency exist in a single sample and it is not in the midrange of expression (around 0.5) where important splits between positive and negative cells are often made.

E.4.3 Eigenvectors

Thanks to that we explicitly model component shapes we can find patterns among the shapes by studying the eigenvectors of the sample component covariance matrices, as in Fig. S4.

E.5 Parameters and convergence for ASPIRE

As recommended, we first standardize the pooled data and then use the parameter values $s = 150 \log(d+1)/d$, $m = d+2$, $\kappa_0 = 0.05$ and $\alpha = \gamma = 1$. To decide κ_i we tried four different recommended values, $\{0.1, 0.25, 0.5, 1\}$. The highest mean likelihood during the production iterations was obtained

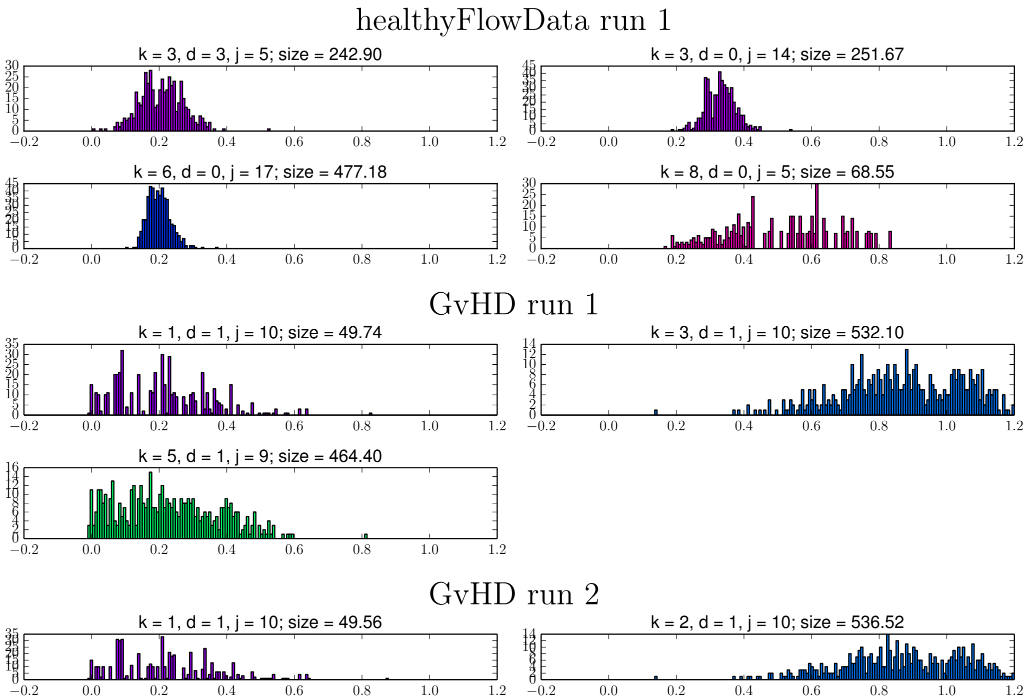

Figure S3: Histograms of quantiles of soft clusters in one dimension. Only dimension–cluster combinations which gives dip tests below 0.28 are shown. Evaluating these is part of the quality control and all the above have been seen as acceptable. Even if there are tendencies of bimodality it can be accepted when it seems likely that the cluster consists of a single population based on the expression.

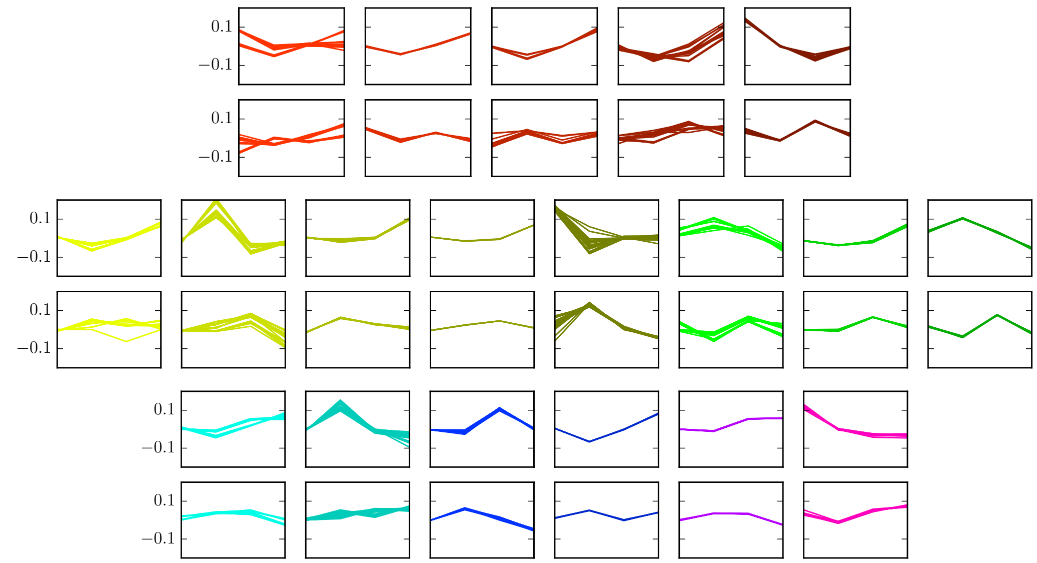

Figure S4: The two first eigenvectors scaled by their corresponding eigenvalues of the 19 active components in the first accepted run for the healthyFlowData dataset. For most components the eigenvectorsj—i.e. the shapes—are very similar across samples, but we can for example also see that for some components there are two groups of shapes.

for $\kappa_i = 0.1$ for both healthyFlowData and GvHD (see Fig. S5), thus we used results from this run as the final results. However, we observed that the likelihood increased monotonically when decreasing κ_i, so for healthyFlowData we also explored additional, smaller values of κ_i, namely 0.05, 0.25 and 0.01. We noted a continued increase in mean likelihood and noted that this was accompanied by a decrease in the number of latent components and an increase in the number of mixture components corresponding to each latent component. For $\kappa_i = 0.01$, essentially all data points ($> 99.99\%$) were assigned to the two largest latent components. This led us to stick to the value $\kappa_i = 0.1$.

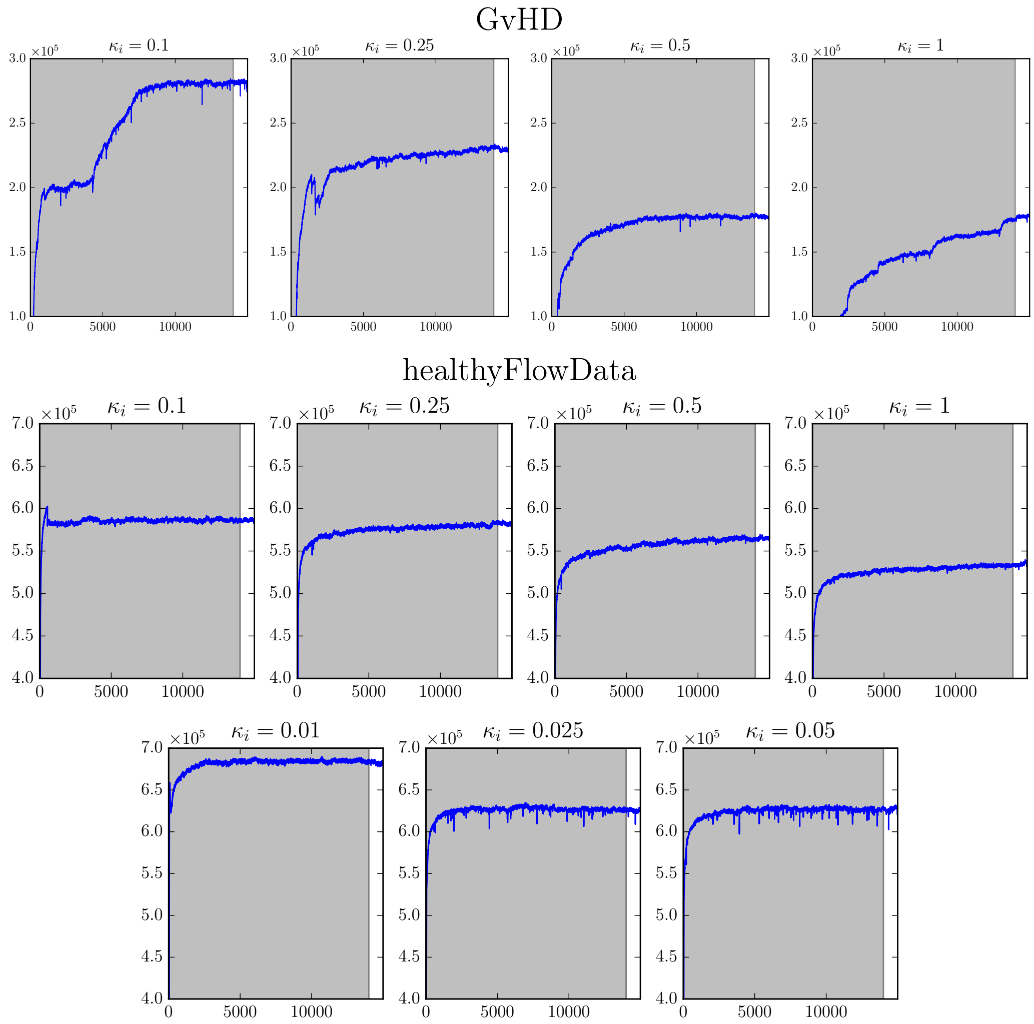

Figure S5: Trace plot of likelihoods for ASPIRE runs with different κ_i. The shaded areas show burn-in iterations.

E.6 GvHD scatterplots

E.7 Individual GMM models with EM for healthyFlow-Data

The variation between flow cytometry samples is systematized in the hierarchical model, results of this can be seen in Fig. 12 and Fig. 13 (a). For comparison, we fit Gaussian mixture models using the expectation-maximization algorithm to each flow cytometry sample separately. In this case there are no clear correspondences among the mixture components between samples, as seen in Fig. S7. When the data set was studied previously with an algorithm matching populations found by separate analysis of the samples, this was only done with a coarse partition of the cell measurements, with four cell populations [9].

References

[1] Robert, C., Casella, G.: Monte Carlo Statistical Methods. Springer Texts in Statistics. Springer, New York (2004)

[2] Roberts, G.O., Rosenthal, J.S.: Examples of adaptive MCMC. Journal of Computational and Graphical Statistics **18**(2), 349–367 (2009)

[3] Green, P.J.: Reversible jump Markov chain Monte Carlo computation and Bayesian model determination. Biometrika **82**(4), 711–732 (1995)

[4] Richardson, S., Green, P.J.: On Bayesian analysis of mixtures with an unknown number of components (with discussion). Journal of the Royal Statistical Society: Series B (Statistical Methodology) **59**(4), 731–792 (1997)

[5] Fukunaga, K.: Introduction to Statistical Pattern Recognition. Academic press, San Diego (1990)

[6] Hartigan, J.A., Hartigan, P.M.: The dip test of unimodality. The Annals of Statistics, 70–84 (1985)

[7] Fisher, R.A.: The use of multiple measurements in taxonomic problems. Annals of eugenics **7**(2), 179–188 (1936)

[8] Hennig, C.: Methods for merging Gaussian mixture components. Advances in Data Analysis and Classification **4**(1), 3–34 (2010)

[9] Azad, A., Khan, A., Rajwa, B., Pyne, S., Pothen, A.: Classifying immunophenotypes with templates from flow cytometry. In: Proceedings of the International Conference on Bioinformatics, Computational Biology and Biomedical Informatics, p. 256 (2013). ACM

[10] Azad, A.: flowVS: Variance Stabilization in Flow Cytometry (and Microarrays). (2015). R package version 1.1.0.

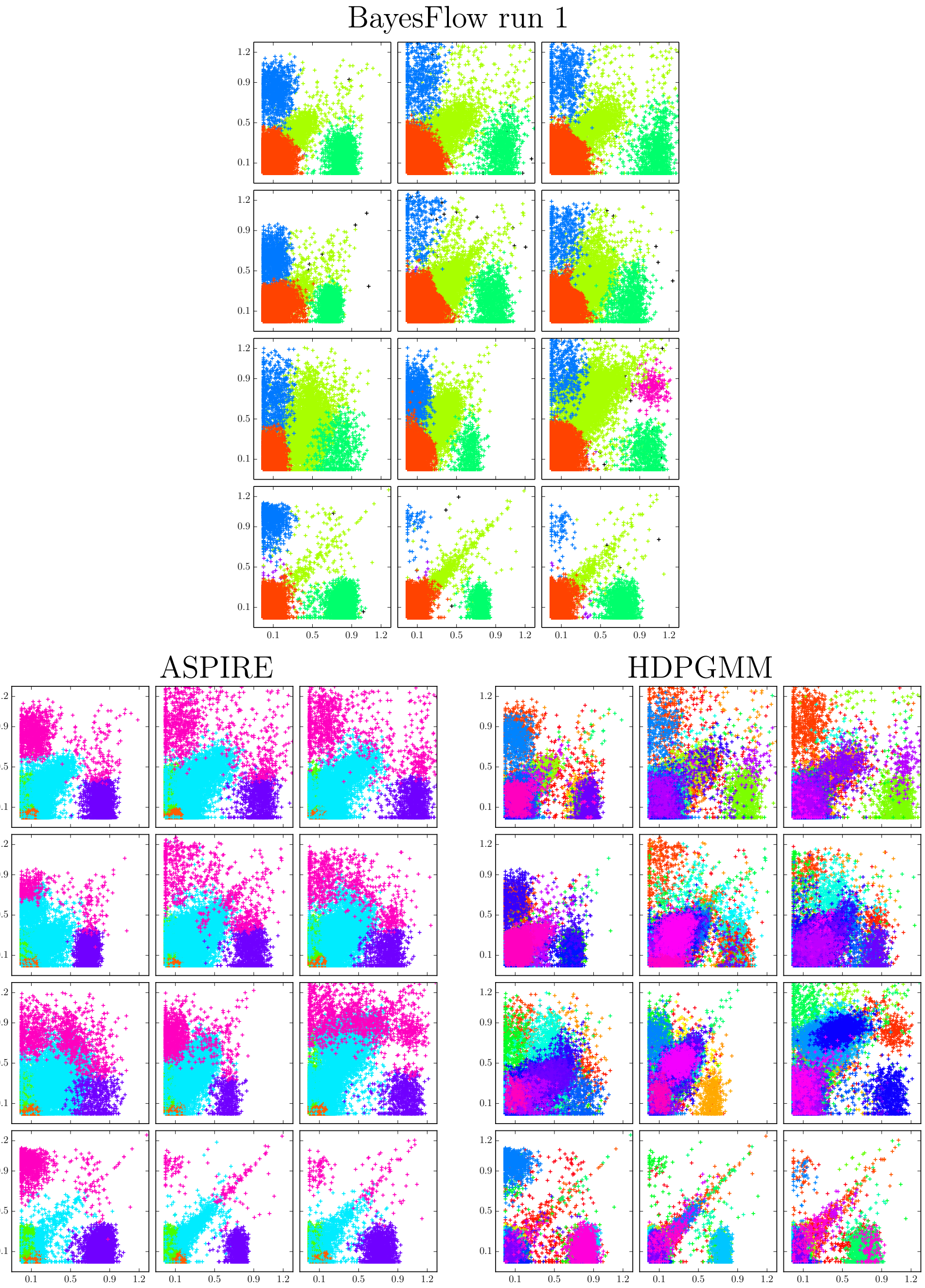

Figure S6: Gated events according to BayesFow run 1, ASPIRE and HDPGMM of the twelve samples in the GvHD dataset, projected onto the two first dimensions.

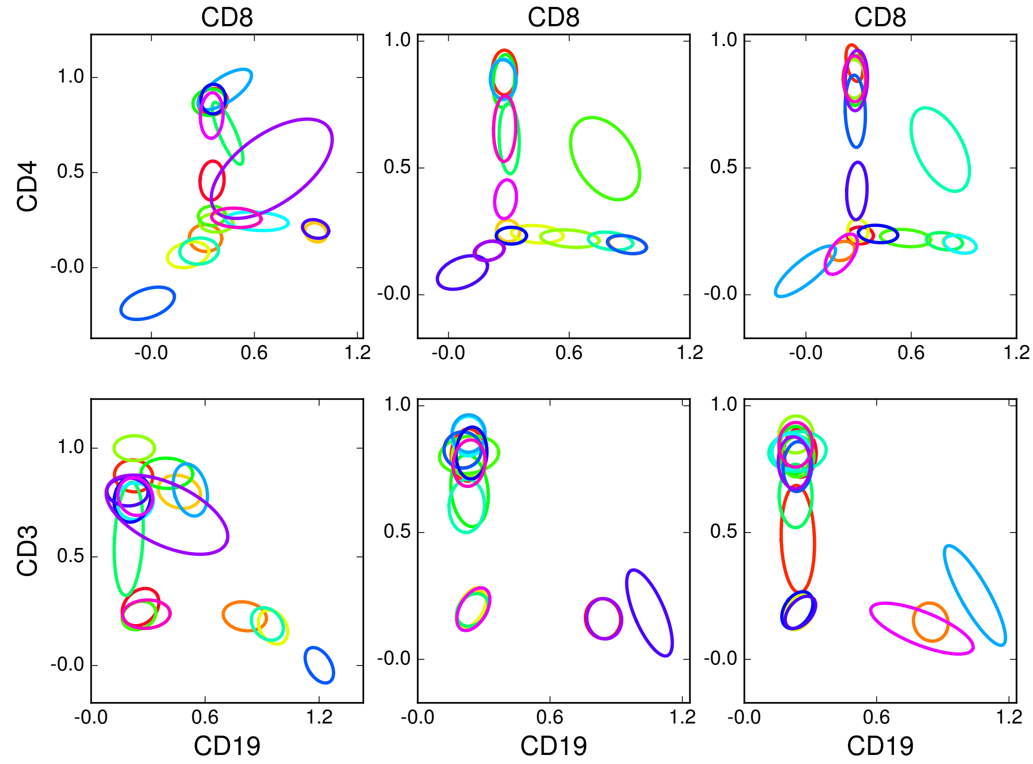

Figure S7: Component parameter representations of inferred mixture components in independent Gaussian mixture models of three flow cytometry samples. The two samples depicted in the two right columns are technical replicates. Note that there is no correspondence between colors between columns.
